# Author Correction: Proteostatic reactivation of the developmental transcription factor TBX3 drives BRAF/MAPK-mediated tumorigenesis

**DOI:** 10.1038/s41467-024-49330-w

**Published:** 2024-06-10

**Authors:** Zhenlei Zhang, Yufan Wu, Jinrong Fu, Xiujie Yu, Yang Su, Shikai Jia, Huili Cheng, Yan Shen, Xianghui He, Kai Ren, Xiangqian Zheng, Haixia Guan, Feng Rao, Li Zhao

**Affiliations:** 1 Department of Thyroid and Neck Oncology, Key Laboratory of Cancer Prevention and Therapy, Tianjin’s Clinical Research Center for Cancer, National Clinical Research Center for Cancer, The Province and Ministry Co-sponsored Collaborative Innovation Center for Medical Epigenetics, Key Laboratory of Immune Microenvironment and Disease (Ministry of Education), Department of Biochemistry and Molecular Biology, School of Basic Medical Sciences, Tianjin Medical University Cancer Institute and Hospital, Tianjin Medical University, Tianjin, China; 2 Department of Endocrinology, Guangdong Provincial People’s Hospital (Guangdong Academy of Medical Sciences), Southern Medical University, Guangzhou, Guangdong China; 3 Department of Pathology, Tianjin Central Hospital of Gynecology and Obstetrics, Tianjin, China; 4 School of Life Sciences, Southern University of Science and Technology, Shenzhen, Guangdong China; 5 Department of General Surgery, Tianjin Medical University General Hospital, Tianjin Medical University, Tianjin, China; 6 Department of Radiation Oncology, Key Laboratory of Cancer Prevention and Therapy, Tianjin’s Clinical Research Center for Cancer, National Clinical Research Center for Cancer, Tianjin Medical University Cancer Institute and Hospital, Tianjin, China; 7 Department of Thyroid and Neck Tumor, Tianjin Medical University Cancer Institute and Hospital, National Clinical Research Center for Cancer, Key Laboratory of Cancer Prevention and Therapy, Tianjin’s Clinical Research Center for Cancer, Tianjin, China; 8 Department of Thyroid and Neck Tumor, Key Laboratory of Cancer Prevention and Therapy, Tianjin’s Clinical Research Center for Cancer, National Clinical Research Center for Cancer, The Province and Ministry Co-sponsored Collaborative Innovation Center for Medical Epigenetics, Key Laboratory of Immune Microenvironment and Disease (Ministry of Education), Department of Biochemistry and Molecular Biology, School of Basic Medical Sciences, Tianjin Medical University Cancer Institute and Hospital, Tianjin Medical University, Tianjin, China

**Keywords:** Tumour biomarkers, Oncogenes, Ubiquitylation, Cell lineage

Correction to: *Nature Communications* 10.1038/s41467-024-48173-9, published online 15 May 2024

In this article, the affiliation details for Li Zhao were incorrectly given as ‘Department of Thyroid and Neck Oncology, Key Laboratory of Cancer Prevention and Therapy, Tianjin’s Clinical Research Center for Cancer, National Clinical Research Center for Cancer, The Province and Ministry Co-sponsored Collaborative Innovation Center for Medical Epigenetics, Key Laboratory of Immune Microenvironment and Disease (Ministry of Education), Department of Biochemistry and Molecular Biology, School of Basic Medical Sciences, Tianjin Medical University Cancer Institute and Hospital, Tianjin Medical University, Tianjin, China’ but should have been ‘Department of Thyroid and Neck Tumor, Key Laboratory of Cancer Prevention and Therapy, Tianjin’s Clinical Research Center for Cancer, National Clinical Research Center for Cancer, The Province and Ministry Co-sponsored Collaborative Innovation Center for Medical Epigenetics, Key Laboratory of Immune Microenvironment and Disease (Ministry of Education), Department of Biochemistry and Molecular Biology, School of Basic Medical Sciences, Tianjin Medical University Cancer Institute and Hospital, Tianjin Medical University, Tianjin, China’. In addition, the affiliation details for Xiangqian Zheng were given incorrectly as ‘Department of Thyroid and Neck Oncology, Tianjin Medical University Cancer Institute and Hospital, National Clinical Research Center for Cancer; Key Laboratory of Cancer Prevention and Therapy, Tianjin’s Clinical Research Center for Cancer, Tianjin, China.’ And should have been ‘Department of Thyroid and Neck Tumor, Tianjin Medical University Cancer Institute and Hospital, National Clinical Research Center for Cancer, Key Laboratory of Cancer Prevention and Therapy, Tianjin’s Clinical Research Center for Cancer, Tianjin, China.

