# Supplementary material for: Proteostatic reactivation of the developmental transcription factor TBX3 drives BRAF/MAPK-mediated tumorigenesis
Source: Nat Commun. 2024 May 15;15:4108. doi: 10.1038/s41467-024-48173-9 (PMC11096176; doi:10.1038/s41467-024-48173-9)
Supplement: Supplementary file 1 — Supplementary Information [file 41467_2024_48173_MOESM1_ESM.pdf]

Proteostatic reactivation of the developmental transcription factor TBX3 drives BRAF/MAPK-mediated tumorigenesis

Zhenlei Zhang[1,8], Yufan Wu[1,8], Jinrong Fu[2,8], Xiujie Yu[3], Yang Su[4], Shikai Jia[1], Huili Cheng[1], Yan Shen[3], Xianghui He[5], Kai Ren[6], Xiangqian Zheng[7], Haixia Guan[2]*, Feng Rao[4]*, Li Zhao[1]*

1. Department of Thyroid and Neck Oncology, Key Laboratory of Cancer Prevention and Therapy, Tianjin's Clinical Research Center for Cancer, National Clinical Research Center for Cancer, The Province and Ministry Co-sponsored Collaborative Innovation Center for Medical Epigenetics, Key Laboratory of Immune Microenvironment and Disease (Ministry of Education), Department of Biochemistry and Molecular Biology, School of Basic Medical Sciences, Tianjin Medical University Cancer Institute and Hospital, Tianjin Medical University, Tianjin, China.
2. Department of Endocrinology, Guangdong Provincial People's Hospital (Guangdong Academy of Medical Sciences), Southern Medical University, Guangzhou, Guangdong, China.
3. Department of Pathology, Tianjin Central Hospital of Gynecology and Obstetrics, Tianjin, China.
4. School of Life Sciences, Southern University of Science and Technology, Shenzhen, Guangdong, China
5. Department of General Surgery, Tianjin Medical University General Hospital, Tianjin Medical University, Tianjin, China.
6. Department of Radiation Oncology, Key Laboratory of Cancer Prevention and Therapy, Tianjin's Clinical Research Center for Cancer, National Clinical Research Center for Cancer, Tianjin Medical University Cancer Institute and Hospital, Tianjin, China.
7. Department of Thyroid and Neck Oncology, Tianjin Medical University Cancer Institute and Hospital, National Clinical Research Center for Cancer; Key Laboratory of Cancer Prevention and Therapy, Tianjin's Clinical Research Center for Cancer, Tianjin, China.

8. These authors contributed equally.

*Correspondence: L.Z., shzhaoli@tmu.edu.cn;

F.R., raof@sustech.edu.cn;

H.G., guanhaixia@gdph.org.cn.

Supplemental Figures

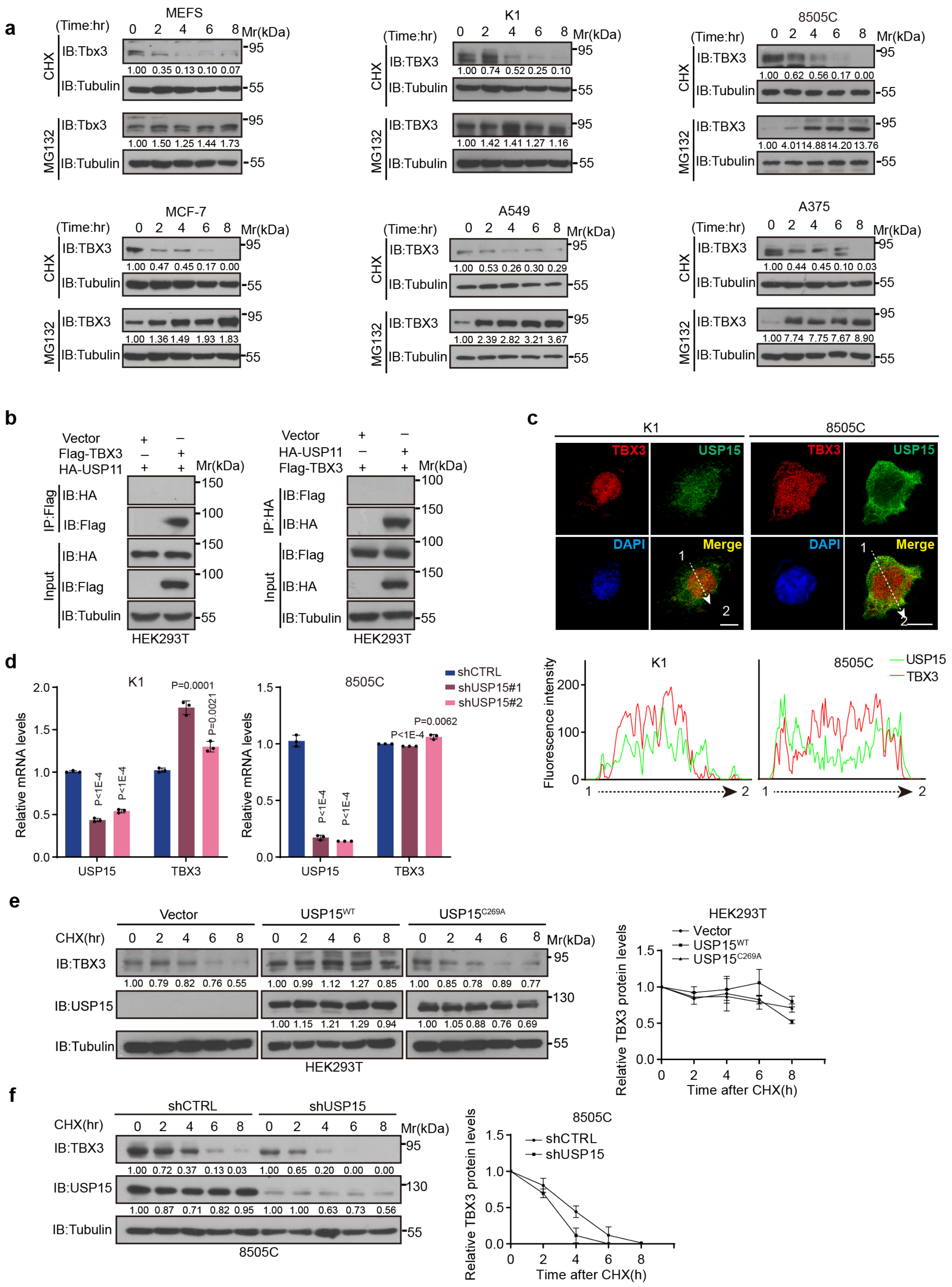

Supplementary Fig.1 USP15 elevates TBX3 stability. a MEFs, thyroid cancer cell K1, 8505C, breast cancer cell MCF-7, lung adenocarcinoma cell A549, melanoma cell A375 lysates were treated with 100 μg/ml CHX (Cycloheximide), 20 μM MG132

for indicated hours and immunoblotting (IB) were used to detect TBX3 expression. **b** Co-IP were performed to examine the interaction between over-expressed HA-USP11 and Flag-TBX3 in HEK293T cells. **c** Immunofluorescence (IF) was performed to validate colocalization of USP15 and TBX3 in K1 and 8505C cells. Scale bars, 20 μm. The blow panel showed fluorescence intensities. **d** TBX3 mRNA expression was detected by qRT-PCR after USP15 knock-down in K1 or 8505C cells. **e** Half-lives of TBX3 were analyzed after wild-type USP15 ($USP15^{WT}$) or mutant USP15 ($USP15^{C269A}$) over-expression. **f** Half-lives of TBX3 was analyzed after USP15 knock-down in 8505C cells. n = 3 biological independent samples. Data are shown as the mean ± s.d (**e**, **f**). P values were calculated by unpaired two-tailed Student's t test (**d**). Densitometric analyses of western blot were shown (**a**, **e**, **f**). Uncropped immunoblots and statistical source data are provided in Source Data.

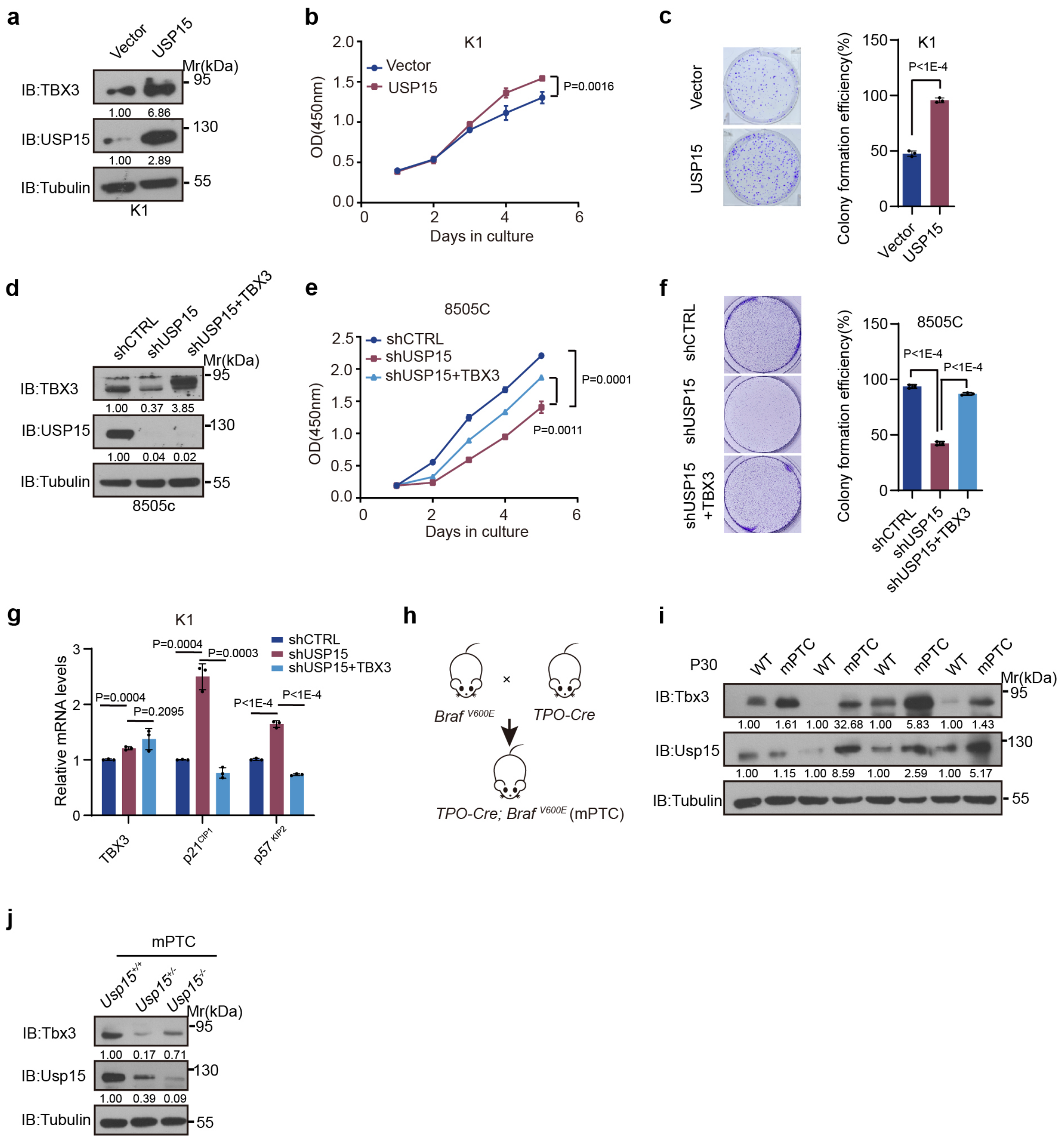

Supplementary Fig.2 USP15 promotes BRAF^{V600E}-induced tumor growth in a TBX3-dependent manner. **a** TBX3 expression was detected after USP15 over-expression in K1 cells. **b, c** K1 cells with USP15 over-expression were subjected to cell growth assays of (**b**) CCK8 or (**c**) colony formation. **d** TBX3 expression was detected after USP15 knocked-down with or without TBX3 over-expression in 8505C cells. **e**, **f** 8505C cells with USP15 knocked-down with or without TBX3 over-expression were subjected to cell growth assays of (**e**) CCK8 or (**f**) colony formation. **g** RT-PCR analysis of TBX3, p21^{CIP1}, p57^{KIP2} on subcutaneous tumors formed from K1 cells, with USP15 knock-down with or without TBX3 over-expression. **h** The transgenic mice crossing strategy of mPTC (*TPO-Cre;*

Braf^{V600E}). **i** IB analysis of Tbx3 and Usp15 in thyroid tissues from WT or mPTC mice at P30. **j** IB analysis of Tbx3 and Usp15 in mPTC, mPTC/Usp15$^{+/-}$, mPTC/Usp15$^{-/-}$ mice at P30. n = 3 biological independent samples (**a**-**g**, **i**-**j**). Representative images are shown (**b**, **c**, **e**, **f**). Data are shown as the mean ± s.d (**c, f**). P values were calculated by unpaired two-tailed Student's t test (**b**, **c**, **e**, **f**, **g**). Densitometric analyses of western blot were shown (**a**, **d**, **i**, **j**). Uncropped immunoblots and statistical source data are provided in Source Data.

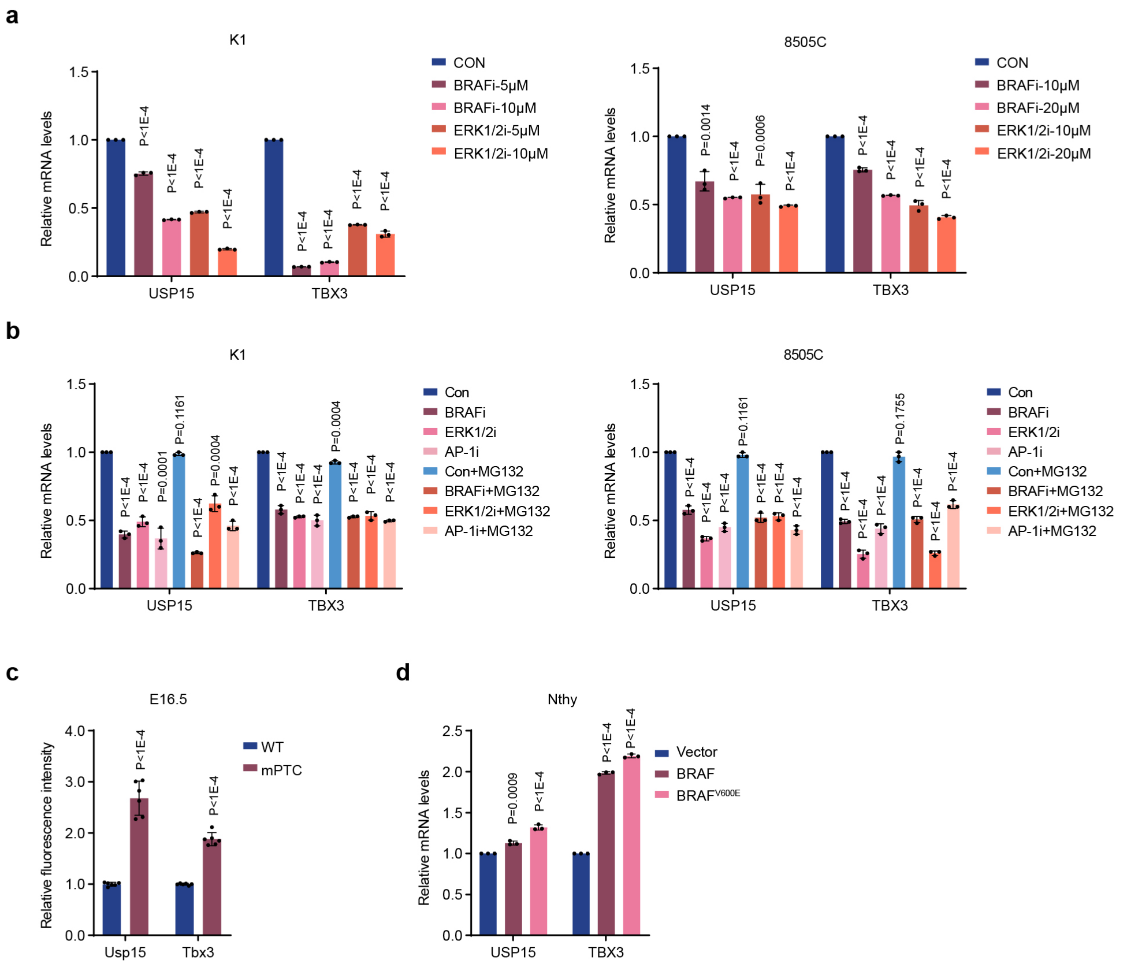

Supplementary Fig.3 USP15 is transcriptionally upregulated during BRAF^{V600E}-induced tumorigenesis. a USP15 and TBX3 mRNA expression was detected by qRT-PCR in K1 and 8505C cells treated with BRAF inhibitor PLX4032 or ERK1/2 inhibitor SCH772984 for 24 hours. **b** USP15 and TBX3 mRNA expression was detected by qRT-PCR in K1 and 8505C cells treated with BRAF inhibitor PLX4032 or ERK1/2 inhibitor SCH772984 for 24 hours, then treated with MG132 (20 μM) for 8 hours. **c** Relative fluorescence intensities for Usp15 or Tbx3 in WT or mPTC E16.5 thyroid tissues from Fig. **4f**. (n = 6) **d** USP15 and TBX3 mRNA expression was detected by qRT-PCR after BRAF^{WT} or BRAF^{V600E} over-expression in Nthy-ori 3-1 cells. n = 3 biological independent samples (**a**, **b**, **d**). Data are shown as the mean ± s.d (**a-d**). P values were calculated by unpaired two-tailed Student's t test (**a-d**). Statistical source data are provided in Source Data.

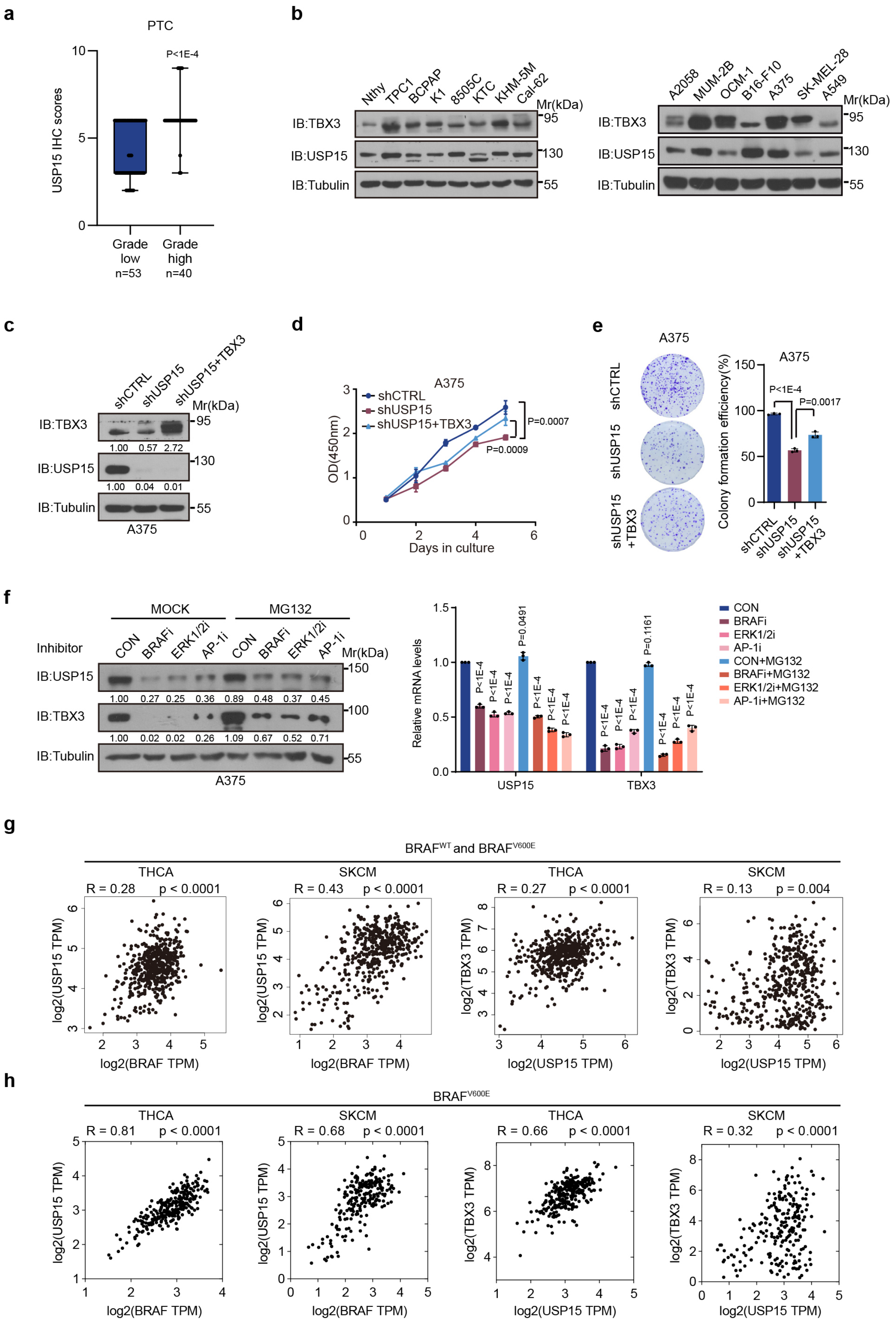

Supplementary Fig.4 TBX3 correlates positively with USP15 in BRAF^{V600E} tumors. a PTC specimens from Figure **5c** with TNM I were stratified into Grade low group and TNM II/III specimens into Grade high group according to the AJCC

Cancer Staging Manual 8th Edition. USP15 IHC staining intensities were plotted between Grade low and Grade high group. Grade low group: minima = 2, maxima = 6, centre = 3. Grade high group: minima = 3, maxima = 9, centre = 6. **b** TBX3, USP15 expressions were detected across different types of tumor cells. Nthy-ori 3-1 (Nthy) was normal human thyroid cell line. K1, BCPAP, KTC were PTC cell lines with $BRAF^{V600E}$-mutation, and TPC-1 was PTC cell line with RET/PTC1 rearrangement ($BRAF^{WT}$). 8505C, KHM-5M were $BRAF^{V600E}$-mutated ATC cell lines, and Cal-62 was RAS-mutated ATC cell line. A2058, A375, and SK-MEL-28 were $BRAF^{V600E}$-mutated melanoma cell lines, and B16-F10 was $BRAF^{WT}$ mouse melanoma cell line. OCM-1 was $BRAF^{V600E}$-mutated uveal melanoma cell line, and MUB-2B was $BRAF^{WT}$ uveal melanoma cell line. MCF-7 was breast cancer cell line, and A549 was KRAS-mutated/$p53^{WT}$ lung adenocarcinoma cell line **c** TBX3 expression was detected in USP15 knocked-down A375 cells with or without TBX3 re-expression. **d**, **e** USP15 knocked-down A375 cells with or without TBX3 over-expression were subjected to cell growth assays of (**d**) CCK8 or (**e**) colony formation. **f** USP15 and TBX3 protein and mRNA expression was detected by IB or qRT-PCR in A375 cells treated with BRAF inhibitor PLX4032 or ERK1/2 inhibitor SCH772984 for 24 hours, and then treated with MG132 (20 μM) for 8 hours. **g** Correlation analysis between USP15 and BRAF or TBX3 and USP15 in THCA and SKCM from TCGA databases ($BRAF^{WT}$ and $BRAF^{V600E}$) was peformed by Pearson correlation. **h** Correlation analysis between TBX3 and USP15 in THCA and SKCM $BRAF^{V600E}$ mutated cases from TCGA databases was peformed by Pearson correlation. Densitometric analyses of western blot were shown (**c**, **f**). n = 3 biological independent samples (**b**-**f**). Representative images are shown (**e**). Data are shown as the mean ± s.d (**a**, **d**, **e**, **f**). P values were calculated by unpaired two-tailed Student's t test (**a**, **d**, **e**, **f**). Uncropped immunoblots and statistical source data are provided in Source Data.

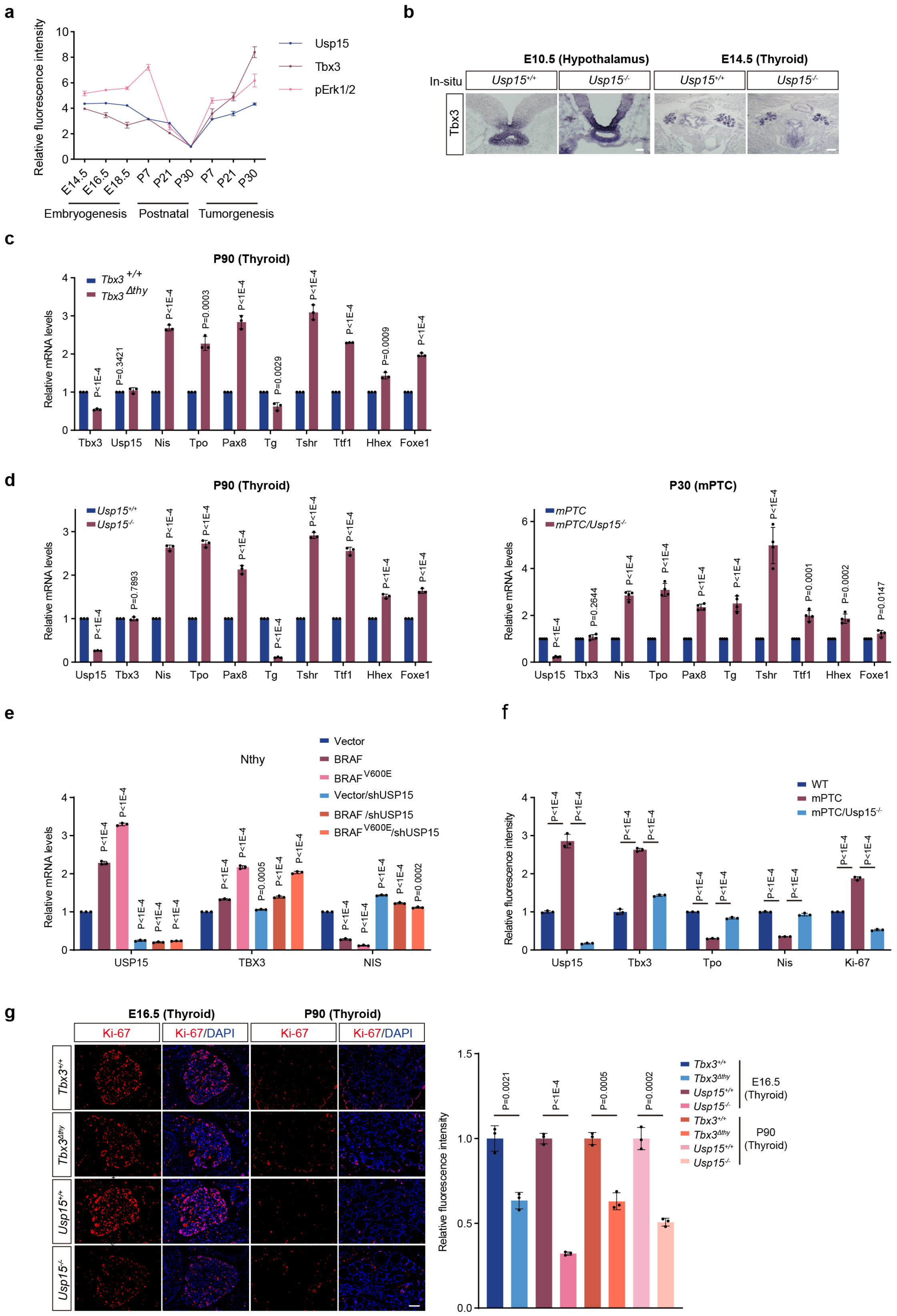

Supplementary Fig.5 USP15 and TBX3 defines lowly differentiated embryonic progenitor and cancerous cell states. a The relative fluorescence intensities of Usp15, Tbx3 and p-Erk1/2 in Fig. **6a**. **b** In-situ hybridization of Tbx3 on sections of

E10.5 hypothalamus (Scale bars, 50 μm) and E14.5 thyroid (Scale bars, 100 μm) from wild-type (*Usp15*$^{+/+}$) or Usp15^{null} mutant mice (*Usp15*$^{-/-}$) (n = 3). **c**, **d** Usp15, Tbx3 and thyroid differentiation (TD) genes mRNA expression was detected by qRT-PCR in thyroid tissues of *Tbx3*$^{+/+}$ or *Tbx3*$^{\Delta thy}$ at P90 (n = 3), *Usp15*$^{+/+}$ or *Usp15*$^{-/-}$ (n = 3) at P90 and mPTC or mPTC/Usp15$^{-/-}$ (n = 4) at P30. **e** USP15, TBX3 and NIS mRNA expression was detected by qRT-PCR after BRAF^{WT} or BRAF^{V600E} over-expression with or without USP15 knock-down in Nthy-ori 3-1 cells (n = 3). **f** The relative fluorescence intensities of Tg, Tpo, Nis and Ki-67 in Fig.**7g**. **g** IF staining of Ki-67 on transverse sections of E16.5 and P90 thyroid tissues from *Tbx3*$^{+/+}$ or *Tbx3*$^{\Delta thy}$ (n = 3), *Usp15*$^{+/+}$ or *Usp15*$^{-/-}$ (n = 3). Scale bars, 50 μm. The right panel showed relative fluorescence intensities. Representative images are shown (**b**, **g**). Data are shown as the mean ± s.d (**a**, **c**, **d**, **e**, **f**, **g**). P values were calculated by unpaired two-tailed Student's t test (**a**, **c**, **d**, **e**, **f**, **g**). Statistical source data are provided in Source Data.
