# Supplementary material for: Proteostatic reactivation of the developmental transcription factor TBX3 drives BRAF/MAPK-mediated tumorigenesis
Source: Nat Commun. 2024 May 15;15:4108. doi: 10.1038/s41467-024-48173-9 (PMC11096176; doi:10.1038/s41467-024-48173-9)
Supplement: Supplementary file 3 — Description of Additional Supplementary Files [file 41467_2024_48173_MOESM3_ESM.pdf]

Description of Additional Supplementary Files

Supplementary Datasets

1. **Supplementary Data 1.** List of all primer sequences used in this paper.
2. **Supplementary Data 2.** List of the associated proteins from Mass spectrometry analysis of HEK293T cells when TBX3 over-expressed.

Supplementary Videos

1. **Supplementary Video 1.** Representative video showing 3D image of Duolink negative control in K1 cells, related to **Fig. 1d**.
2. **Supplementary Video 2.** Representative video showing 3D image of Duolink TBX3 in K1 cells, related to **Fig. 1d**.
3. **Supplementary Video 3.** Representative video showing 3D image of Duolink USP15 in K1 cells, related to **Fig. 1d**.
4. **Supplementary Video 4.** Representative video showing 3D image of Duolink TBX3-USP15 in K1 cells, related to **Fig. 1d**.
