# Supplementary material for: Proteostatic reactivation of the developmental transcription factor TBX3 drives BRAF/MAPK-mediated tumorigenesis
Source: Nat Commun. 2024 May 15;15:4108. doi: 10.1038/s41467-024-48173-9 (PMC11096176; doi:10.1038/s41467-024-48173-9)
Supplement: Supplementary file 10 — Reporting Summary [file 41467_2024_48173_MOESM10_ESM.pdf]

nature portfolio

Corresponding author(s): Li Zhao
Last updated by author(s): Feb 20, 2024

Reporting Summary

Nature Portfolio wishes to improve the reproducibility of the work that we publish. This form provides structure for consistency and transparency in reporting. For further information on Nature Portfolio policies, see our Editorial Policies and the Editorial Policy Checklist.

Statistics

For all statistical analyses, confirm that the following items are present in the figure legend, table legend, main text, or Methods section.

n/a	Confirmed	
☐	☒	The exact sample size (n) for each experimental group/condition, given as a discrete number and unit of measurement
☐	☒	A statement on whether measurements were taken from distinct samples or whether the same sample was measured repeatedly
☐	☒	The statistical test(s) used AND whether they are one- or two-sided<br>*Only common tests should be described solely by name; describe more complex techniques in the Methods section.*
☒	☐	A description of all covariates tested
☒	☐	A description of any assumptions or corrections, such as tests of normality and adjustment for multiple comparisons
☐	☒	A full description of the statistical parameters including central tendency (e.g. means) or other basic estimates (e.g. regression coefficient) AND variation (e.g. standard deviation) or associated estimates of uncertainty (e.g. confidence intervals)
☐	☒	For null hypothesis testing, the test statistic (e.g. F, t, r) with confidence intervals, effect sizes, degrees of freedom and P value noted<br>*Give P values as exact values whenever suitable.*
☒	☐	For Bayesian analysis, information on the choice of priors and Markov chain Monte Carlo settings
☒	☐	For hierarchical and complex designs, identification of the appropriate level for tests and full reporting of outcomes
☐	☒	Estimates of effect sizes (e.g. Cohen's d, Pearson's r), indicating how they were calculated

Our web collection on statistics for biologists contains articles on many of the points above.

Software and code

Policy information about availability of computer code

Data collection	qRT-PCR data was collected by LightCycler 480 real-time PCR system (Roche) or QuantStudio 1 real-time PCR system (Applied Biosystems) ; Fluorescent images were acquired using Leica THUNDER Imager system or Olympus fluorescence microscopy.
Data analysis	Image J, Graph Pad Prism 9.0 were used for statistical analyses. HTSeq 0.6.0 and DESeq2 1.20.0 were used to analyzed RNA-Seq data with cutoff. Proteome Discoverer 1.3 was used to analyzed Secondary mass spectrometry data.

For manuscripts utilizing custom algorithms or software that are central to the research but not yet described in published literature, software must be made available to editors and reviewers. We strongly encourage code deposition in a community repository (e.g. GitHub). See the Nature Portfolio guidelines for submitting code & software for further information.

Data

Policy information about availability of data

All manuscripts must include a data availability statement. This statement should provide the following information, where applicable:

- Accession codes, unique identifiers, or web links for publicly available datasets
- A description of any restrictions on data availability
- For clinical datasets or third party data, please ensure that the statement adheres to our policy

IP-MS raw data for HEK293T cells were deposited in iProX (IPX0008211001).The RNA-seq data generated in this study have been deposited in the Gene Expression Omnibus (GEO) under the accession GSE255930 available from GEO datasets. GEO Accession Number: GSE166513, TCGA-THCA dataset (https://portal.gdc.cancer.gov/projects/TCGA-THCA) and TCGA-SKCM dataset (https://portal.gdc.cancer.gov/projects/TCGA-SKCM) were used for analyses in this study.

Research involving human participants, their data, or biological material

Policy information about studies with human participants or human data. See also policy information about sex, gender (identity/presentation), and sexual orientation and race, ethnicity and racism.

Reporting on sex and gender	*Use the terms sex (biological attribute) and gender (shaped by social and cultural circumstances) carefully in order to avoid confusing both terms. Indicate if findings apply to only one sex or gender; describe whether sex and gender were considered in study design; whether sex and/or gender was determined based on self-reporting or assigned and methods used.*<br>*Provide in the source data disaggregated sex and gender data, where this information has been collected, and if consent has been obtained for sharing of individual-level data; provide overall numbers in this Reporting Summary. Please state if this information has not been collected.*<br>*Report sex- and gender-based analyses where performed, justify reasons for lack of sex- and gender-based analysis.*
Reporting on race, ethnicity, or other socially relevant groupings	*Please specify the socially constructed or socially relevant categorization variable(s) used in your manuscript and explain why they were used. Please note that such variables should not be used as proxies for other socially constructed/relevant variables (for example, race or ethnicity should not be used as a proxy for socioeconomic status).*<br>*Provide clear definitions of the relevant terms used, how they were provided (by the participants/respondents, the researchers, or third parties), and the method(s) used to classify people into the different categories (e.g. self-report, census or administrative data, social media data, etc.)*<br>*Please provide details about how you controlled for confounding variables in your analyses.*
Population characteristics	*Describe the covariate-relevant population characteristics of the human research participants (e.g. age, genotypic information, past and current diagnosis and treatment categories). If you filled out the behavioural & social sciences study design questions and have nothing to add here, write "See above."*
Recruitment	*Describe how participants were recruited. Outline any potential self-selection bias or other biases that may be present and how these are likely to impact results.*
Ethics oversight	*Identify the organization(s) that approved the study protocol.*

Note that full information on the approval of the study protocol must also be provided in the manuscript.

Field-specific reporting

Please select the one below that is the best fit for your research. If you are not sure, read the appropriate sections before making your selection.

☒ Life sciences ☐ Behavioural & social sciences ☐ Ecological, evolutionary & environmental sciences

For a reference copy of the document with all sections, see nature.com/documents/nr-reporting-summary-flat.pdf

Life sciences study design

All studies must disclose on these points even when the disclosure is negative.

Sample size	The sample size for each experiment is specified in the Methods section and figure legends. The sample size was determined by relevant published research or previous experience with similar type of experiments (Zhang P, Guan H, Yuan S, et al. Nat Commun. 2022;13; Li X, Ruan X, Zhang P, et al. Oncogene. 2018;37:2773-2792).
Data exclusions	For tumor formation experiments and mPTC mouse model (Fig. 3c, 3h, 5h), the highest and lowest tumor of each group were excluded from analysis according to the pre-established exclusion criteria.
Replication	For each experiment, all the number of biological replicates are reported in the figure legends.
Randomization	All cell samples were randomly allocated to experimental and control groups. In each mice experimental serie, animals were assigned to various groups in random according to planned age and weight.

Blinding

The subsequent analysis were performed by different operators in each mouse experimental series. The immunohistochemical analysis of the PTC was finished by two pathologists who did not know each other. For the other cell experiments, two investigators performed respectively with different cell lines . And data analysis was blinded to the experimental conditions.

Reporting for specific materials, systems and methods

We require information from authors about some types of materials, experimental systems and methods used in many studies. Here, indicate whether each material, system or method listed is relevant to your study. If you are not sure if a list item applies to your research, read the appropriate section before selecting a response.

Materials & experimental systems

n/a	Involved in the study	
☐	☒	Antibodies
☐	☒	Eukaryotic cell lines
☒	☐	Palaeontology and archaeology
☐	☒	Animals and other organisms
☒	☐	Clinical data
☒	☐	Dual use research of concern
☒	☐	Plants

Methods

n/a	Involved in the study	
☒	☐	ChIP-seq
☒	☐	Flow cytometry
☒	☐	MRI-based neuroimaging

Antibodies

Antibodies used

All antibodies used in this study are stated in Methods.
Anti-Tbx3 antibody Abcam Cat#ab99302
Monoclonal antibody-FLAG ® M2 mouse mAb Sigma Cat#F3165 Clone (DDDDK)
Monoclonal anti-α-tubulin mouse mAb Sigma Cat# T5168 Clone (B-5-1-2)
HA-Tag Rabbit mAb CST Cat#3724 Clone(C29F4)
Ubiquitin (P4D1) Mouse mAb CST Cat#3936
GST-Tag Antibody CST Cat#2622
Polyclonal antibody-USP15 Rabbit BETHYL Cat#A300-923A
USP15 Monoclonal antibody mouse Proteintech Cat#67557-1-Ig Clone (1C9G3)
Anti-Ki-67 antibody Abcam Cat#ab16667 Clone (SP6)
Anti-Tpo antibody Abcam Cat#ab278525 Clone (EPR23574-405)
Anti-Tg antibody Abcam Cat#156008 Clone (EPR9730)
Anti-Nis antibody Thermo Cat#MA5-12308 (FP5A)
Anti-PAX8 antibody Abcam Cat#ab53490 Clone(PAX8R1)
Anti-Gr-1 antibody BioLegend #108401 Clone(RB6-8C5)
Anti-TTF1 antibody Abcam Cat#ab76013 Clone(EP1584Y)

Validation

All antibodies were obtained from commercial sources with reported validation, and we confirmed with negative and positive control before the study.
Abbreviation for species cross reactivity:H-human, M-mouse, R-rat, Hm-hamster, Mk-monkey, Vir-virus, Mi-mink, C-chicken, Dm-Drosophila melanogaster, X-xenopus, Z-zebrafish, B-bovine, Dg-dog, PG-pig, Sc-Saccharomyces cerevisiae, Ce-caenorhabdities elegans, Hr-horse, K-Kanraroo, Su-sea urchin, Ch-Chlamydomonas, S-sheep.
Anti-Tbx3 antibody Abcam Cat#ab99302, H, M, R, C, PMID: 27553211
Monoclonal antibody-FLAG ® M2 mouse mAb Sigma Cat#F3165 Clone DDDDK, all species reactivity, PMID: 19525223
Monoclonal anti-α-tubulin mouse mAb Sigma Cat# T5168, Clone B-5-1-2, M, C, Ch, Mk, H, R, B, Su, PMID:32193381
HA-Tag (C29F4) Rabbit mAb CST Cat#3724, Clone C29F4, all species reactivity, PMID: 34824248
Ubiquitin (P4D1) Mouse mAb CST Cat#3936, all species reactivity, PMID: 34853315
GST-Tag Antibody CST Cat#2622, all species reactivity, PMID: 30150766
Polyclonal antibody-USP15 Rabbit BETHYL Cat#A300-923A, H, M, PMID: 30874560
USP15 Monoclonal antibody mouse Proteintech Cat#67557-1-Ig, Clone 1C9G3, H, M, R, PMID: 33771975
Anti-Ki-67 antibody Abcam Cat#ab16667 Clone SP6, H, M, R, PMID: 34284046
Anti-Tpo antibody Abcam Cat#ab278525, M, R
Anti-Tg antibody Abcam Cat#156008, Clone EPR9730, H, R, M, PMID: 35332119
Anti-Nis antibody Thermo Cat#MA5-12308, Clone FP5A, P, R, H.PMID: 29846633
Anti-PAX8 antibody Abcam Cat#ab53490, Clone PAX8R1, H, R, M, PMID: 32054838
Anti-Gr-1 antibody BioLegend #108401, Clone RB6-8C5, M, PMID: 35332119
Anti-TTF1 antibody Abcam Cat#ab76013, Clone EP1584Y, H, R, M, PMID: 34931663

Eukaryotic cell lines

Policy information about cell lines and Sex and Gender in Research

Cell line source(s)	HEK293T cells (ACS-4500) were purchased from the American Type Culture Collection (ATCC). Thyroid cancer cell lines K1, BCPAP, TPC-1, KTC-1, 8505C, Cal-62, and normal human thyroid cell line Nthy-ori 3-1 (Nthy) were obtained from Tianjin Medical University Cancer Institute and Hospital with STR profiles. Human melanoma cell lines A375, SK-MEL-28, A2058, and mouse melanoma cells B16-F10 were generously provided by Dr Lizhi Hu from Tianjin Medical University. Thyroid cancer cell line KHM-5M (3101HUMSCSP549), human uveal melanoma cell lines OCM-1, MUB-2B, human breast cancer cell line MCF-7 (1101HUM-PUMC000013) and human lung adenocarcinoma cell line A549 (1101HUM-PUMC000002) were obtained from Cell Resource Center, Peking Union Medical College (PCRC).
Authentication	Cell lines have been confirmed with STR Authentication.
Mycoplasma contamination	All cell lines have been tested for mycoplasma and found to be negative.
Commonly misidentified lines (See ICLAC register)	We didn't use commonly misidentified lines.

Animals and other research organisms

Policy information about studies involving animals; ARRIVE guidelines recommended for reporting animal research, and Sex and Gender in Research

Laboratory animals	Animals used in this study were maintained as specific-pathogen free (SPF) mice on a C57BL/6 genetic background, both sexes we used at E13.5-6 months including Tpo-Cre, LSL-BrafV600ECA, Tbx3flox/ flox, Uspl5null mice , Rosa26-mTmG.<br>6-8-week-old female BALB/c nude mice were injected with tumor cells for tumor xenografts .<br>All mice were maintained at normal room temperature with a 12/12h light/ dark cycle and normal ambient humidity.
Wild animals	No wild animals were used.
Reporting on sex	BALB/c nude mice of female, C57BL/6 mice both sexes we used.
Field-collected samples	The study did not involve field-collected samples.
Ethics oversight	All mouse experiment procedures and protocols were evaluated and authorized by the Regulations of Tianjin Laboratory Animal Management and strictly followed the guidelines under the Institutional Animal Care and Use Committee of Tianjin Medical University (Approval number: SYXK-(Jin) 2019-0004).

Note that full information on the approval of the study protocol must also be provided in the manuscript.

Plants

Seed stocks	*Report on the source of all seed stocks or other plant material used. If applicable, state the seed stock centre and catalogue number. If plant specimens were collected from the field, describe the collection location, date and sampling procedures.*
Novel plant genotypes	*Describe the methods by which all novel plant genotypes were produced. This includes those generated by transgenic approaches, gene editing, chemical/radiation-based mutagenesis and hybridization. For transgenic lines, describe the transformation method, the number of independent lines analyzed and the generation upon which experiments were performed. For gene-edited lines, describe the editor used, the endogenous sequence targeted for editing, the targeting guide RNA sequence (if applicable) and how the editor was applied.*
Authentication	*Describe any authentication procedures for each seed stock used or novel genotype generated. Describe any experiments used to assess the effect of a mutation and, where applicable, how potential secondary effects (e.g. second site T-DNA insertions, mosiacism, off-target gene editing) were examined.*
