# Supplementary material for: Proteostatic reactivation of the developmental transcription factor TBX3 drives BRAF/MAPK-mediated tumorigenesis
Source: Nat Commun. 2024 May 15;15:4108. doi: 10.1038/s41467-024-48173-9 (PMC11096176; doi:10.1038/s41467-024-48173-9)

Fig.1b

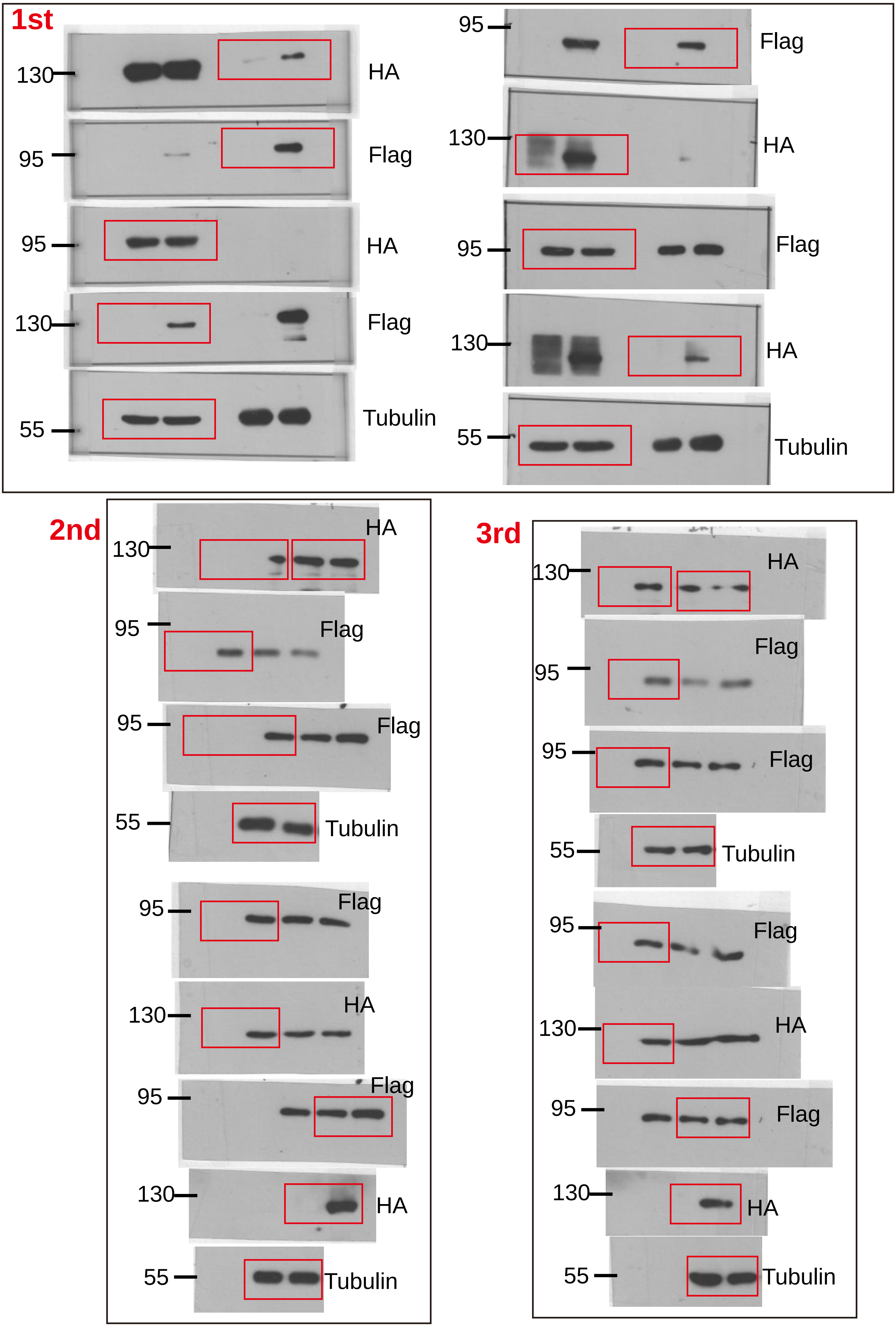
1st
130
HA
95
Flag
95
HA
130
Flag
55
Tubulin
95
Flag
130
HA
95
Flag
130
HA
55
Tubulin
2nd
130
HA
95
Flag
95
Flag
55
Tubulin
95
Flag
130
HA
95
Flag
130
HA
55
Tubulin
3rd
130
HA
95
Flag
95
Flag
55
Tubulin
95
Flag
130
HA
95
Flag
130
HA
55
Tubulin

Fig.1c

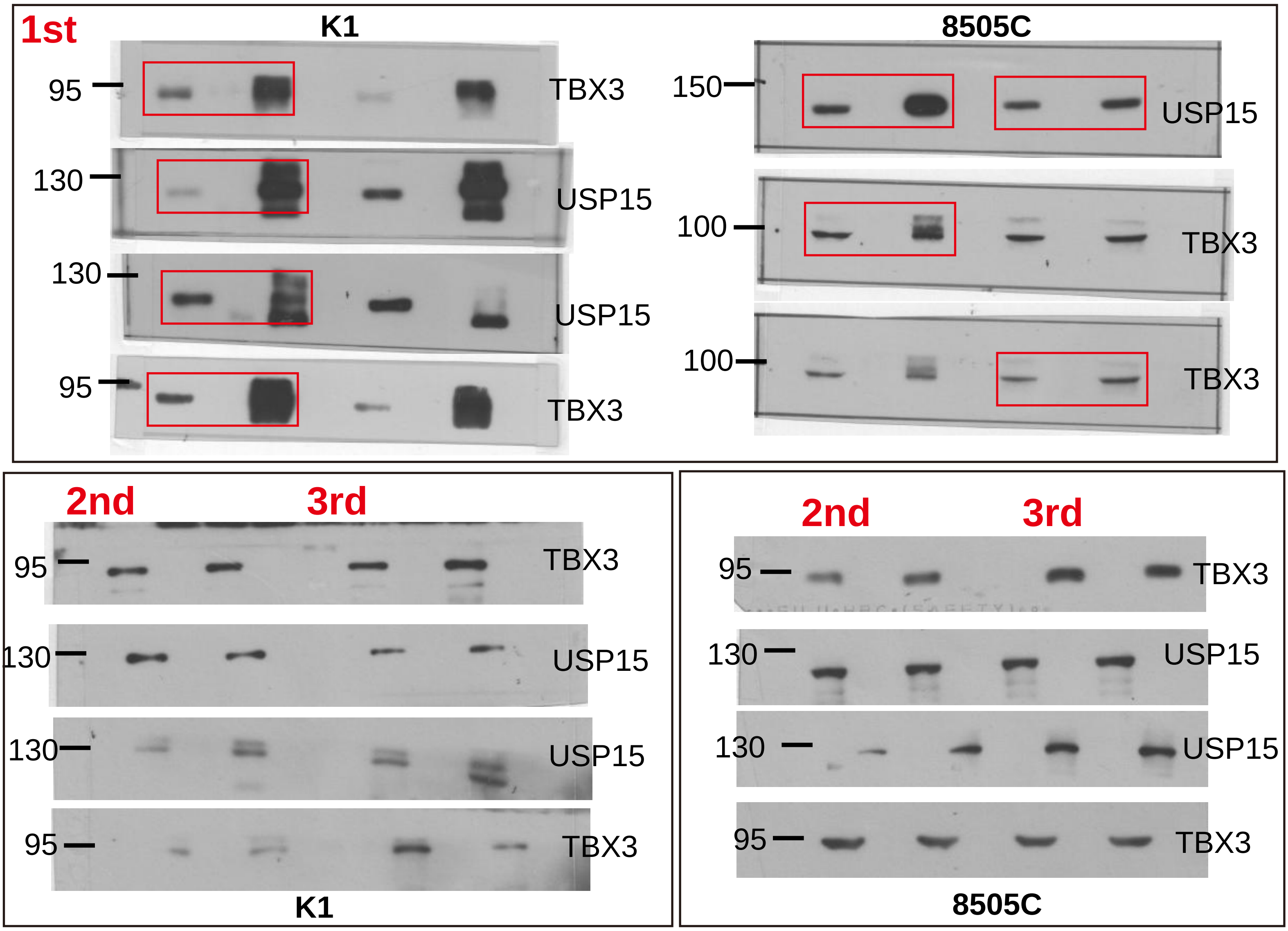
1st
K1
8505C
95
130
130
95
TBX3
USP15
USP15
TBX3
150
100
100
USP15
TBX3
TBX3
2nd
3rd
95
130
130
95
TBX3
USP15
USP15
TBX3
K1
2nd
3rd
95
130
130
95
TBX3
USP15
USP15
TBX3
8505C

Fig.1e

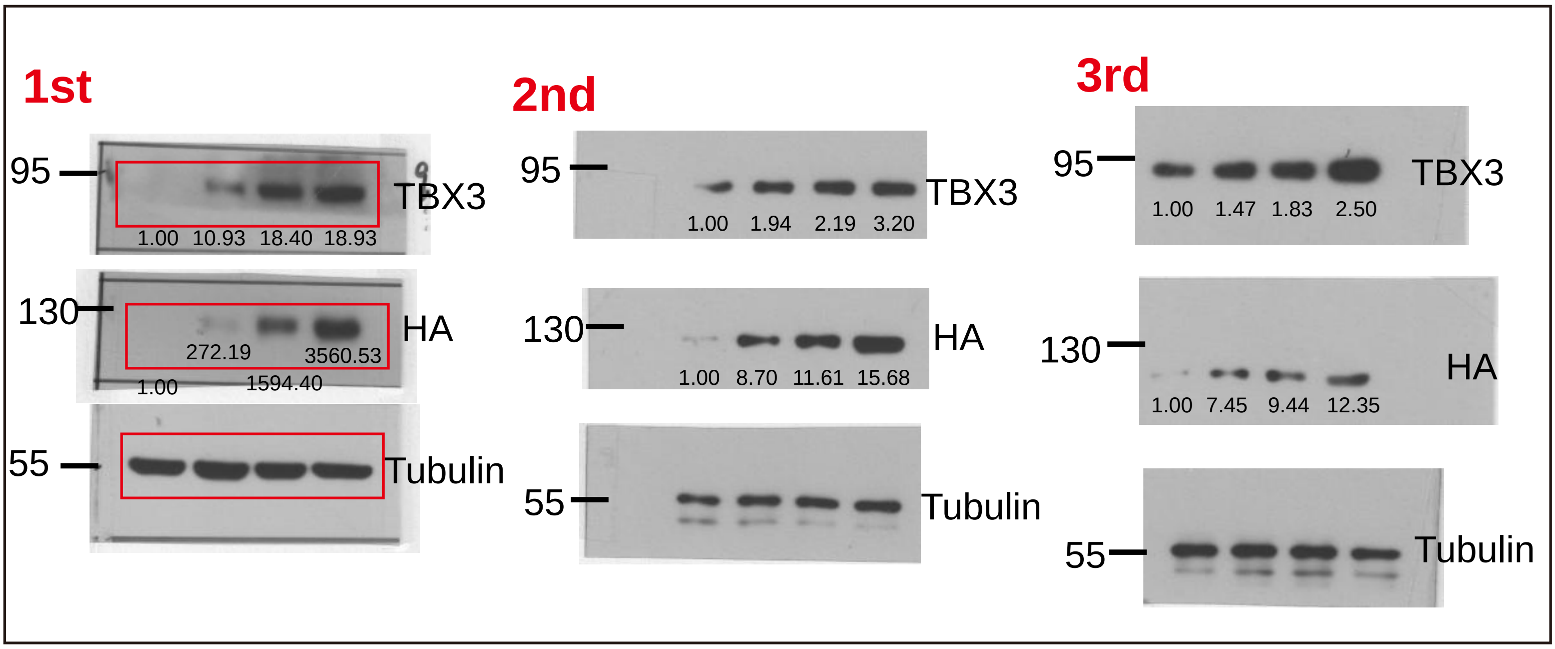
1st
95
TBX3
1.00 10.93 18.40 18.93
130
HA
272.19 3560.53
1.00 1594.40
55
Tubulin
2nd
95
TBX3
1.00 1.94 2.19 3.20
130
HA
1.00 8.70 11.61 15.68
55
Tubulin
3rd
95
TBX3
1.00 1.47 1.83 2.50
130
HA
1.00 7.45 9.44 12.35
55
Tubulin

Fig.1f

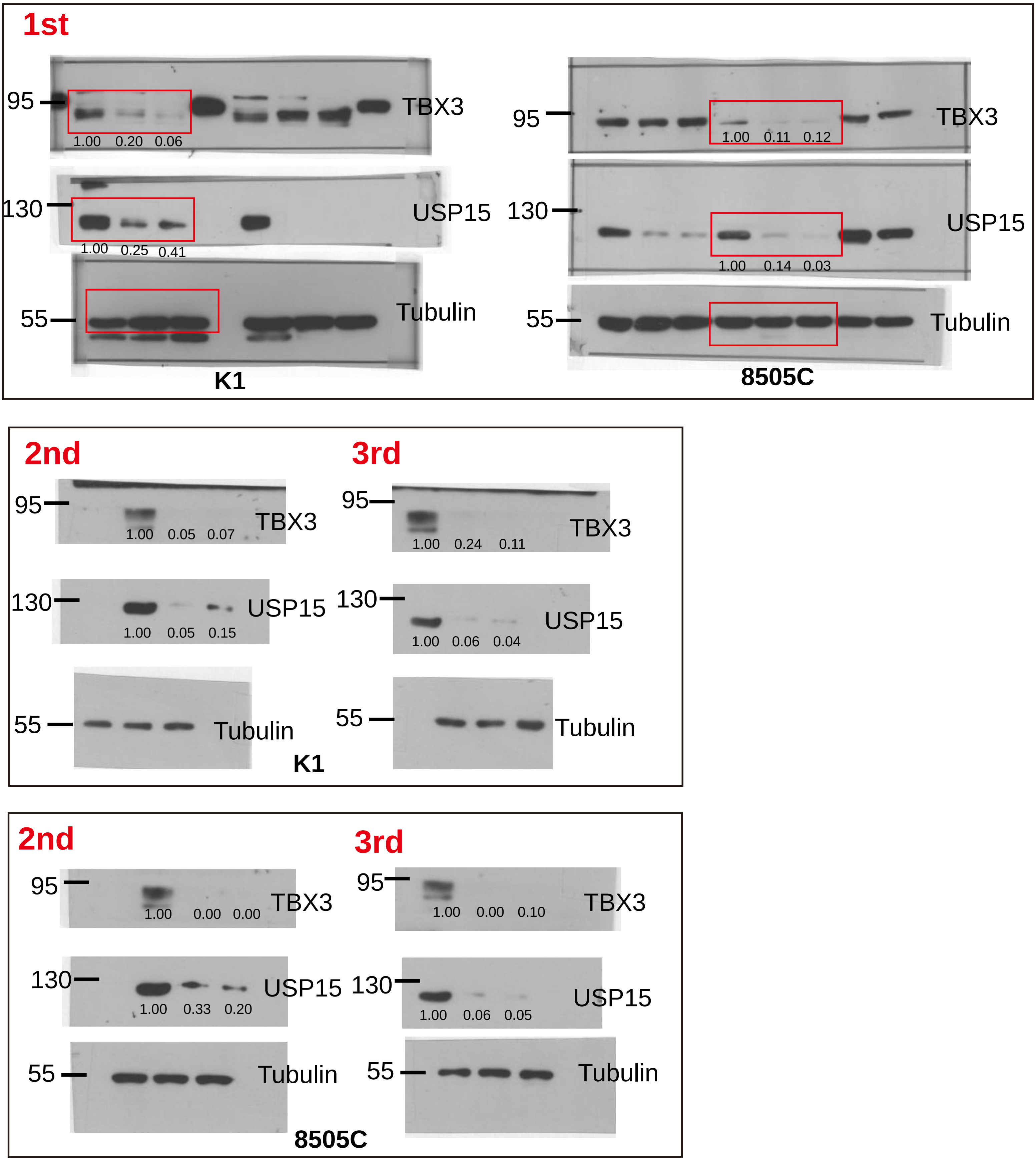
1st
95
TBX3
1.00 0.20 0.06
130
USP15
1.00 0.25 0.41
55
Tubulin
K1
95
TBX3
1.00 0.11 0.12
130
USP15
1.00 0.14 0.03
55
Tubulin
8505C
2nd
95
TBX3
1.00 0.05 0.07
130
USP15
1.00 0.05 0.15
55
Tubulin
K1
3rd
95
TBX3
1.00 0.24 0.11
130
USP15
1.00 0.06 0.04
55
Tubulin
2nd
95
TBX3
1.00 0.00 0.00
130
USP15
1.00 0.33 0.20
55
Tubulin
8505C
3rd
95
TBX3
1.00 0.00 0.10
130
USP15
1.00 0.06 0.05
55
Tubulin

Fig.1g

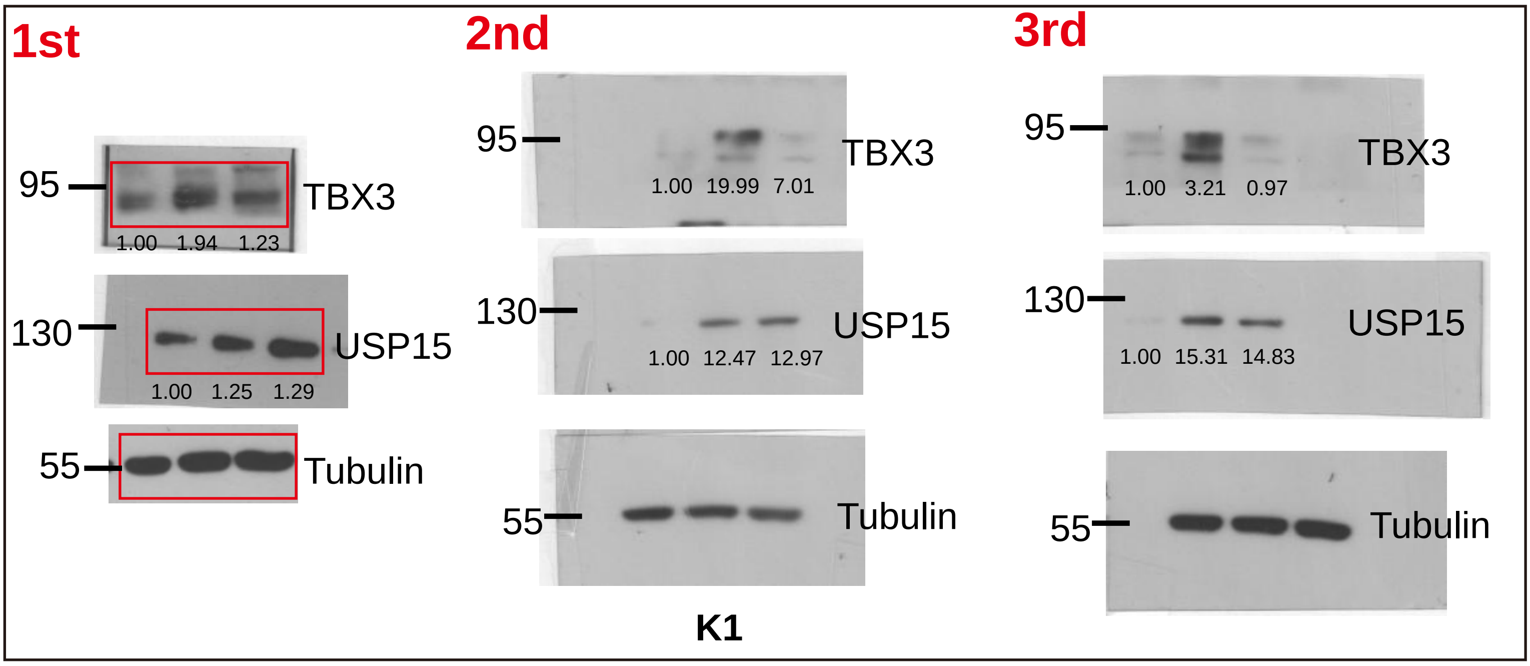
1st
2nd
3rd
95
TBX3
1.00 1.94 1.23
130
USP15
1.00 1.25 1.29
55
Tubulin
1.00 19.99 7.01
1.00 12.47 12.97
1.00 3.21 0.97
1.00 15.31 14.83
K1

Fig.1h

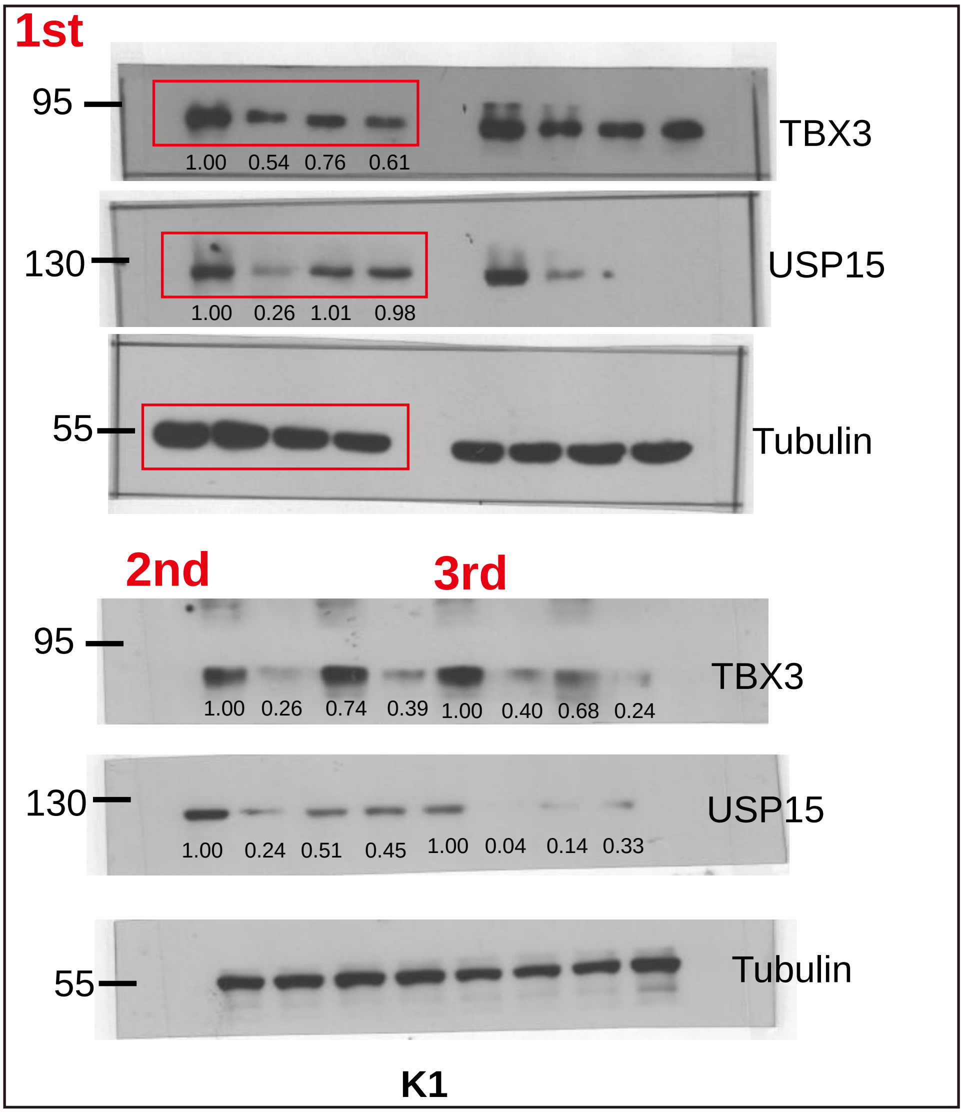
1st
95
TBX3
1.00 0.54 0.76 0.61
130
USP15
1.00 0.26 1.01 0.98
55
Tubulin
2nd
3rd
1.00 0.26 0.74 0.39 1.00 0.40 0.68 0.24
1.00 0.24 0.51 0.45 1.00 0.04 0.14 0.33
K1

Fig.1i

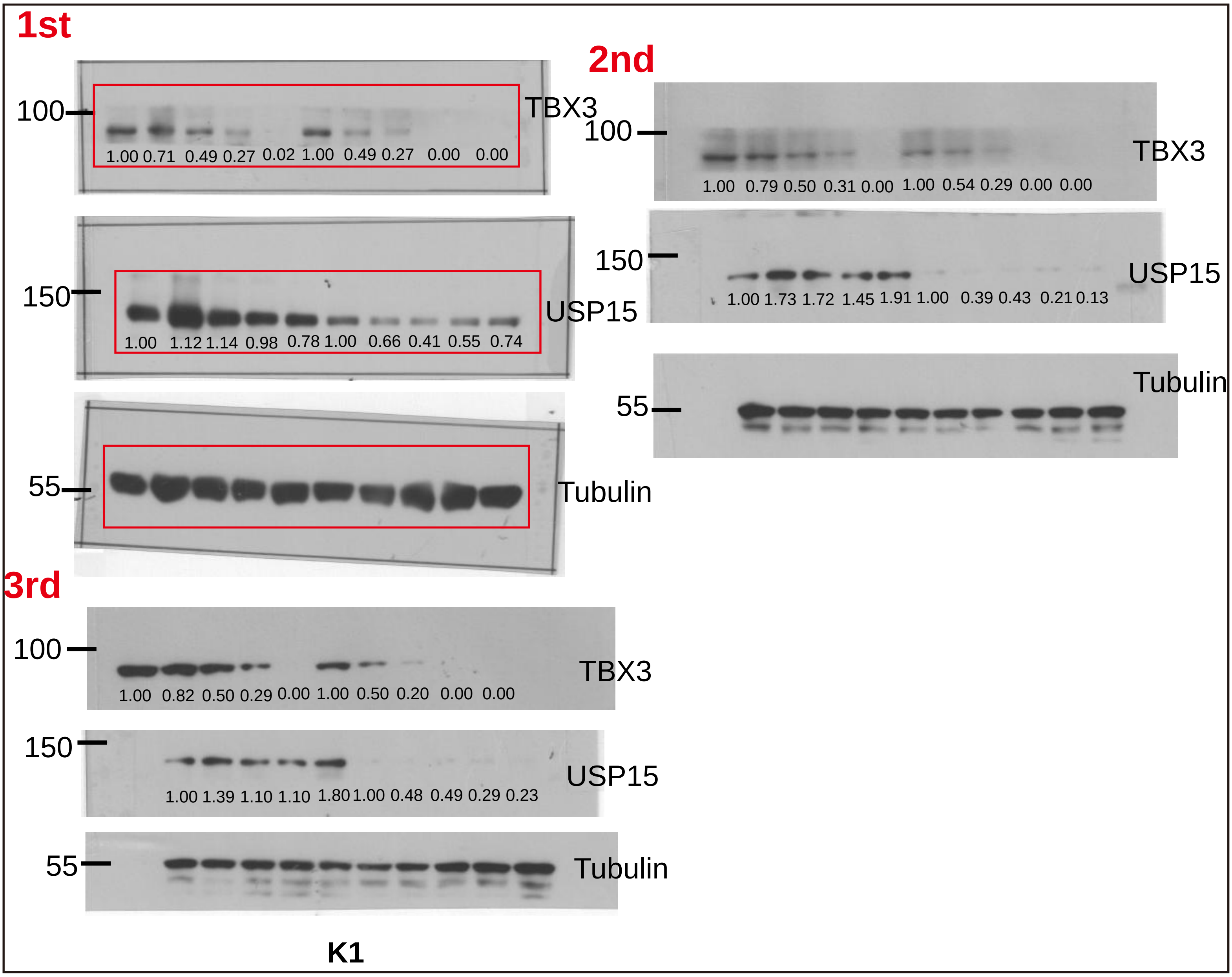
1st
100
TBX3
1.00 0.71 0.49 0.27 0.02 1.00 0.49 0.27 0.00 0.00
150
USP15
1.00 1.12 1.14 0.98 0.78 1.00 0.66 0.41 0.55 0.74
55
Tubulin
2nd
100
TBX3
1.00 0.79 0.50 0.31 0.00 1.00 0.54 0.29 0.00 0.00
150
USP15
1.00 1.73 1.72 1.45 1.91 1.00 0.39 0.43 0.21 0.13
55
Tubulin
3rd
100
TBX3
1.00 0.82 0.50 0.29 0.00 1.00 0.50 0.20 0.00 0.00
150
USP15
1.00 1.39 1.10 1.10 1.80 1.00 0.48 0.49 0.29 0.23
55
Tubulin
K1

Fig.1j

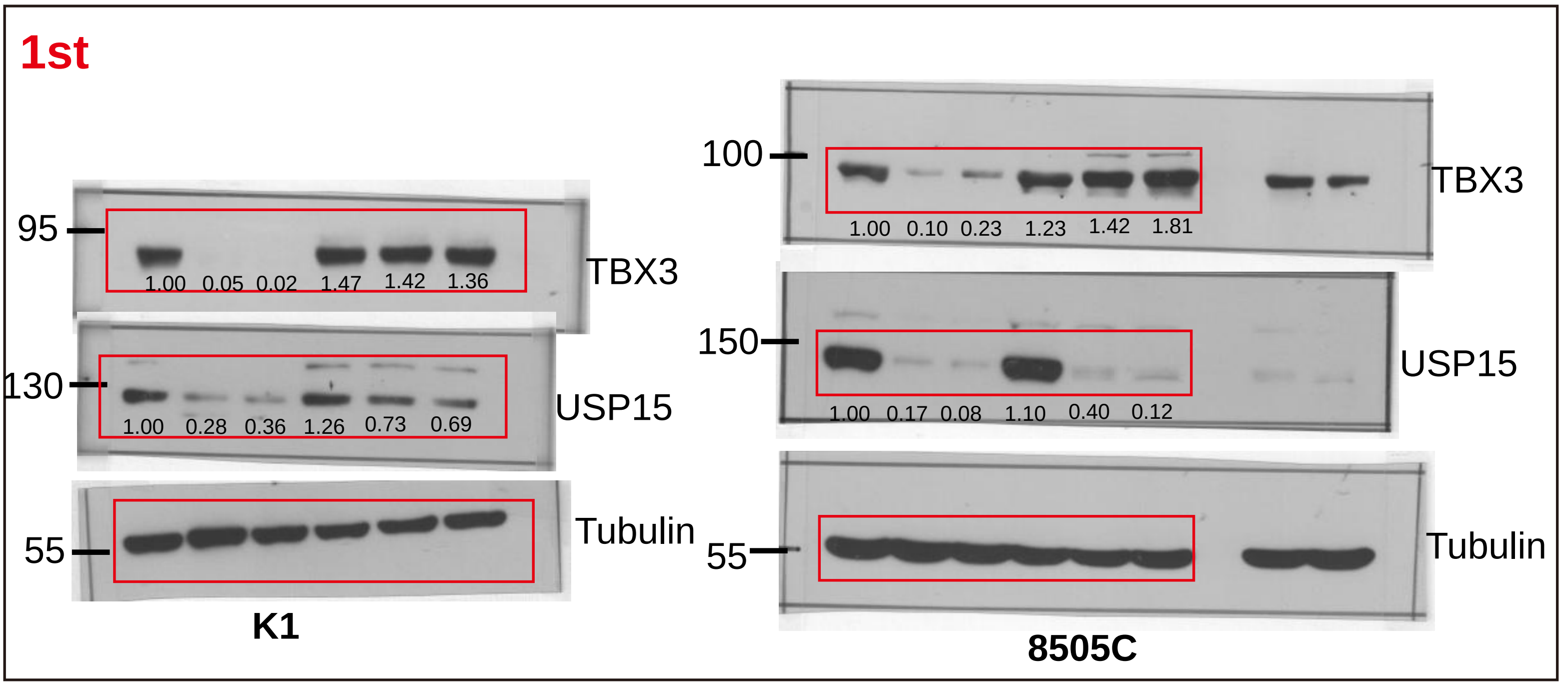
1st
95
TBX3
1.00 0.05 0.02 1.47 1.42 1.36
130
USP15
1.00 0.28 0.36 1.26 0.73 0.69
55
Tubulin
K1
100
TBX3
1.00 0.10 0.23 1.23 1.42 1.81
150
USP15
1.00 0.17 0.08 1.10 0.40 0.12
55
Tubulin
8505C

Fig.1j

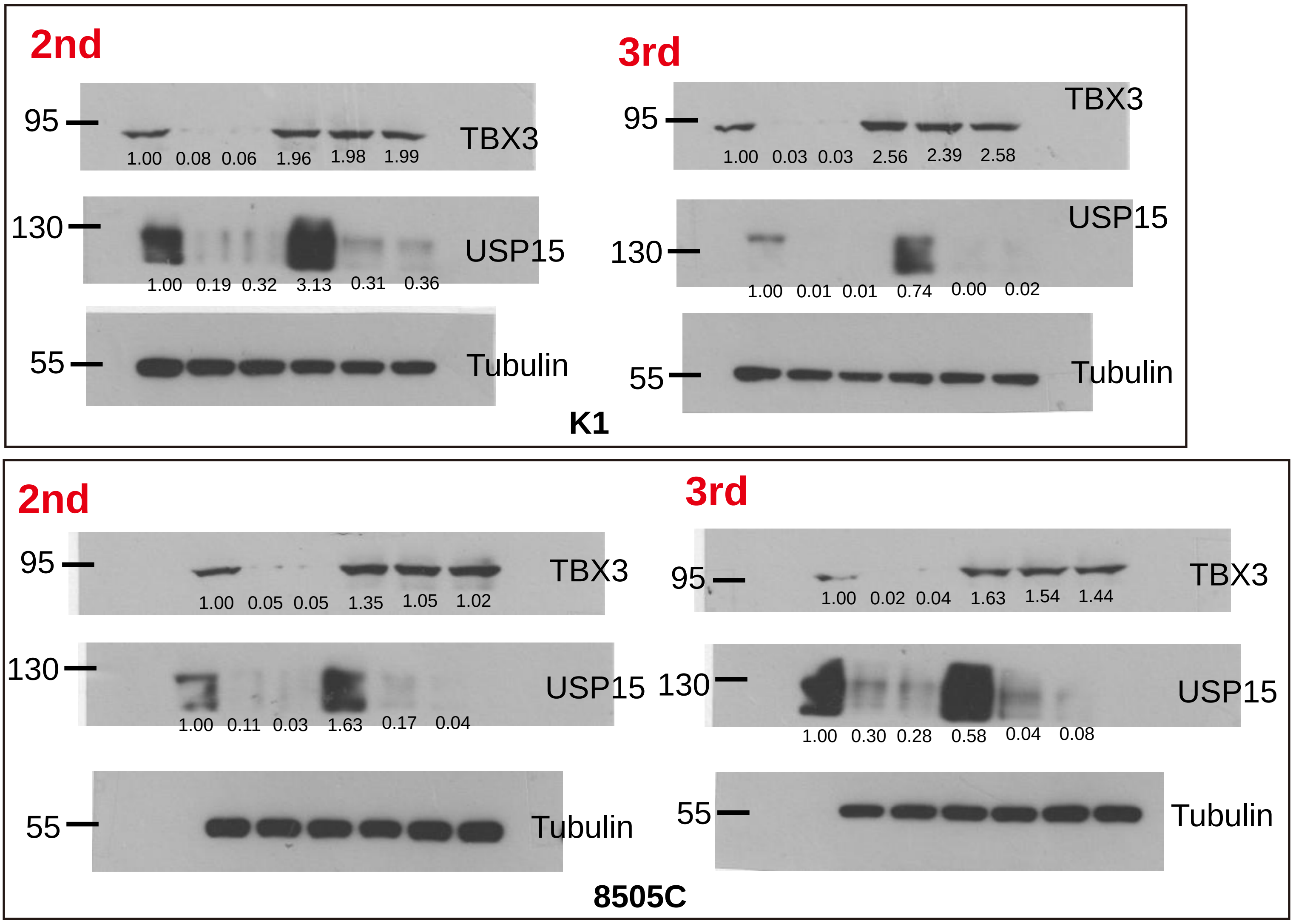
2nd
95
TBX3
1.00 0.08 0.06 1.96 1.98 1.99
130
USP15
1.00 0.19 0.32 3.13 0.31 0.36
55
Tubulin
3rd
95
TBX3
1.00 0.03 0.03 2.56 2.39 2.58
130
USP15
1.00 0.01 0.01 0.74 0.00 0.02
55
Tubulin
K1
2nd
95
TBX3
1.00 0.05 0.05 1.35 1.05 1.02
130
USP15
1.00 0.11 0.03 1.63 0.17 0.04
55
Tubulin
3rd
95
TBX3
1.00 0.02 0.04 1.63 1.54 1.44
130
USP15
1.00 0.30 0.28 0.58 0.04 0.08
55
Tubulin
8505C

Fig.2a

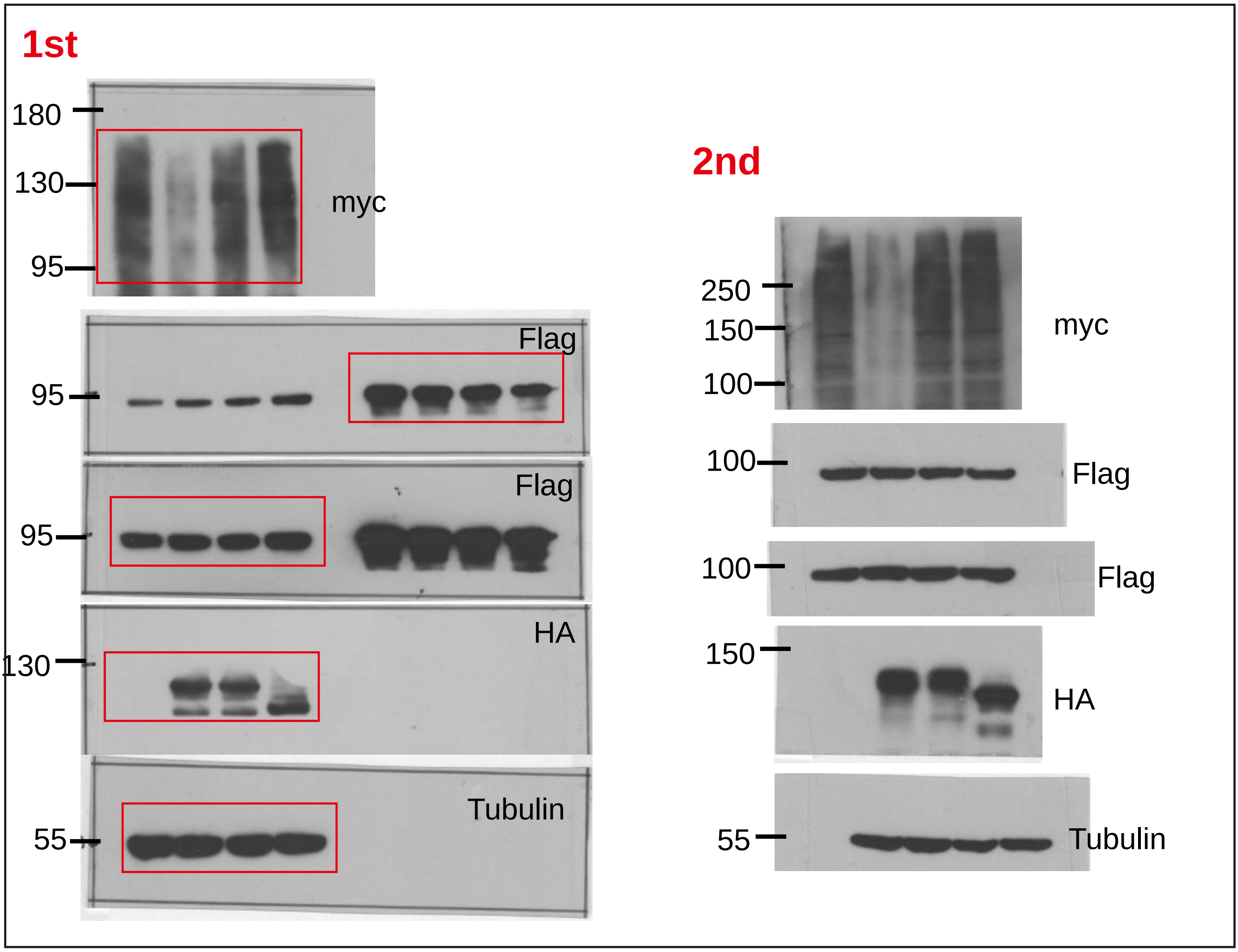
1st
180
130
95
myc
95
Flag
95
Flag
130
HA
55
Tubulin
2nd
250
150
100
myc
100
Flag
100
Flag
150
HA
55
Tubulin

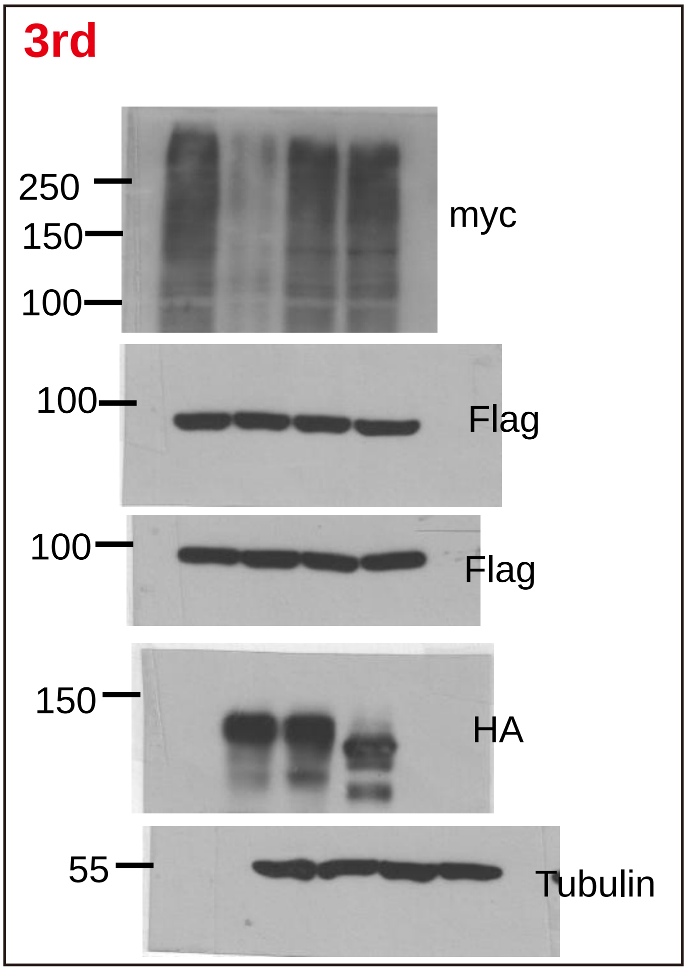
3rd
250
150
100
myc
100
Flag
100
Flag
150
HA
55
Tubulin

Fig.2b

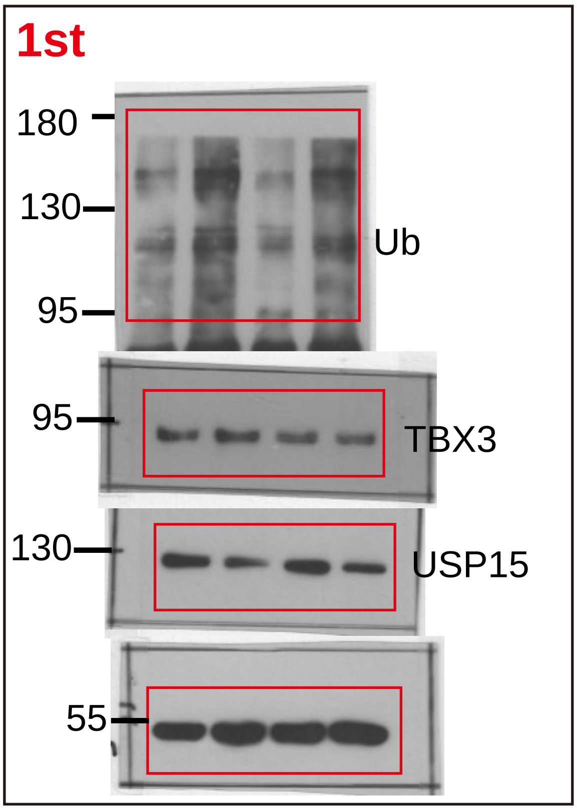
1st
180
130
95
Ub
95
TBX3
130
USP15
55

Fig.2b

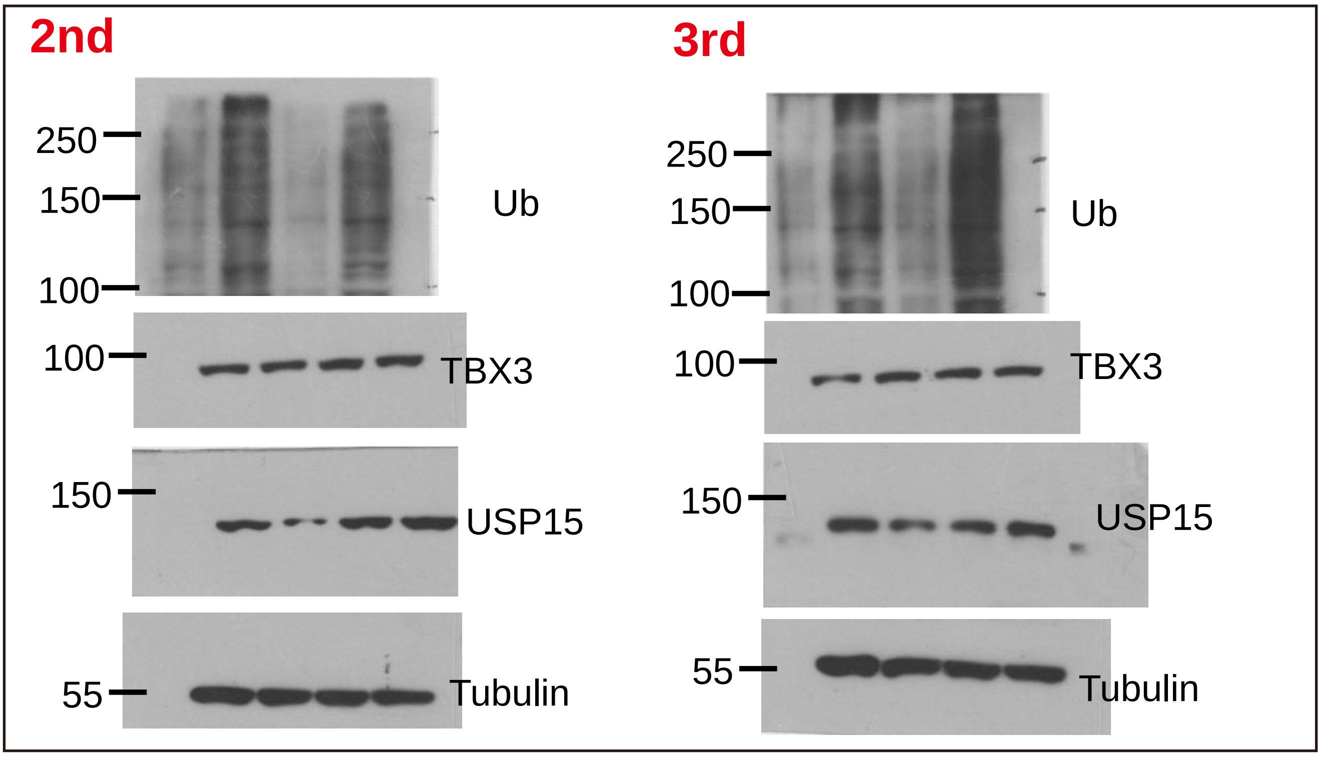

Fig.2c

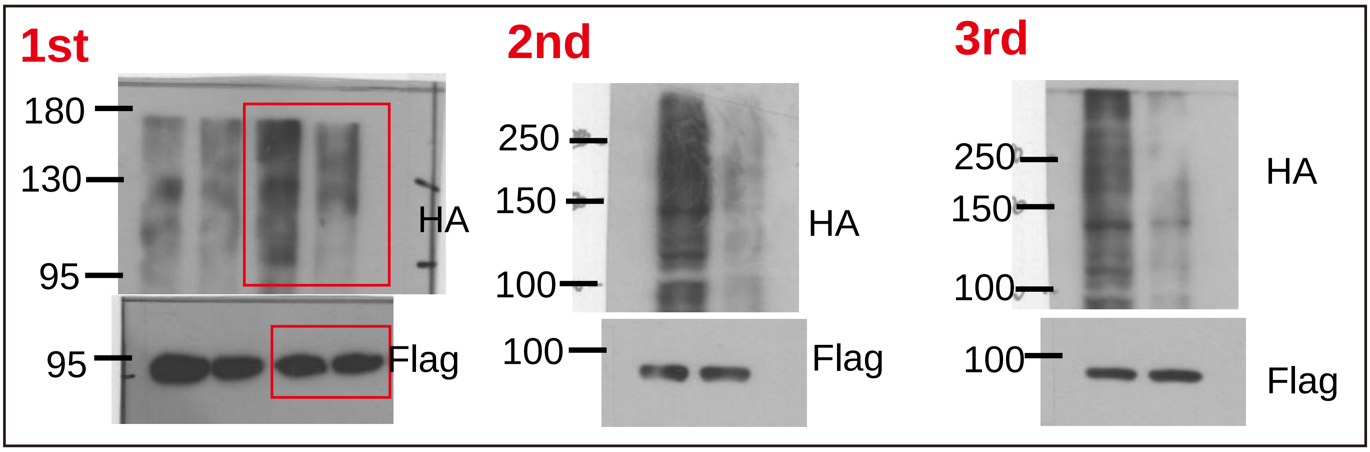

Fig.2d

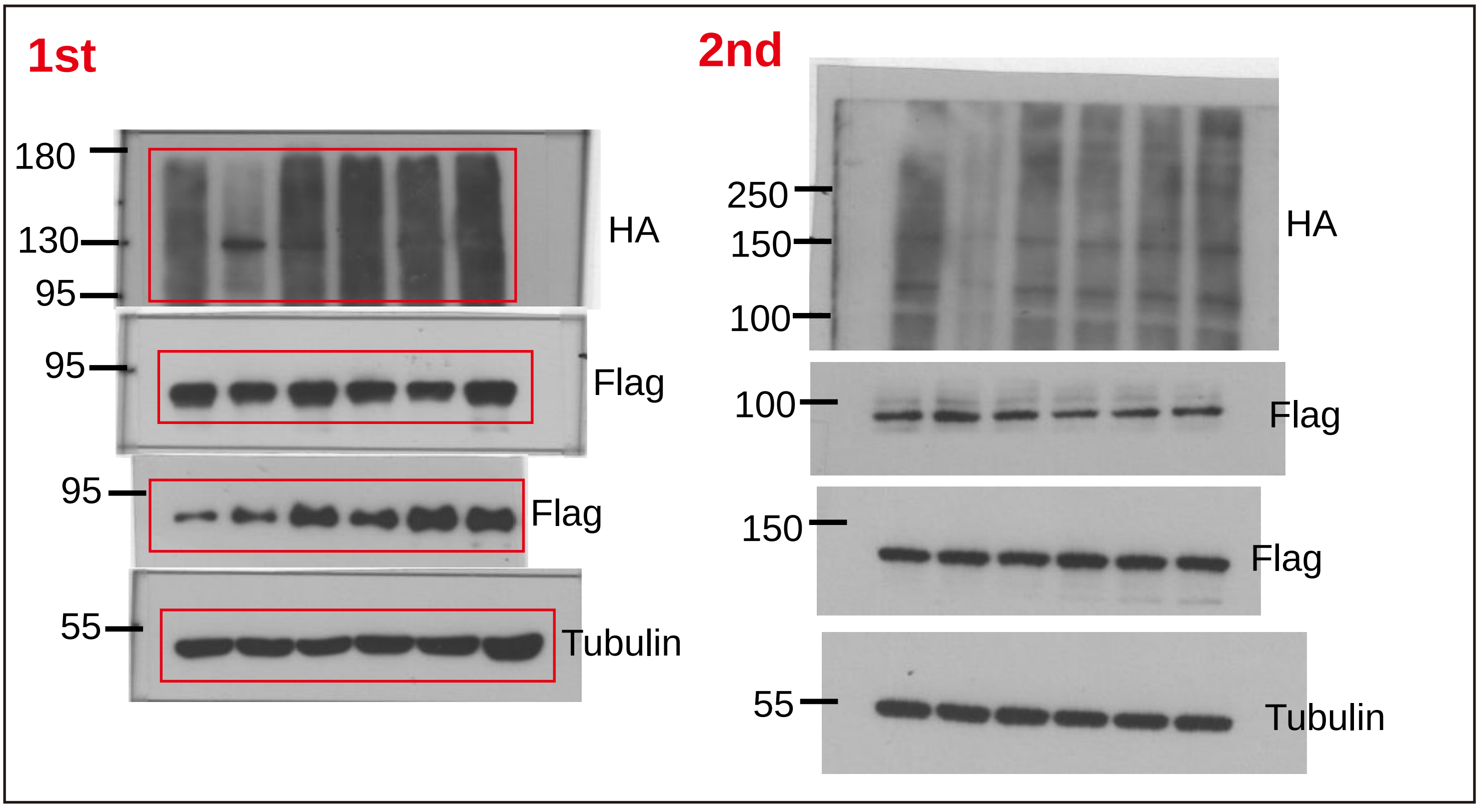

Fig.2d

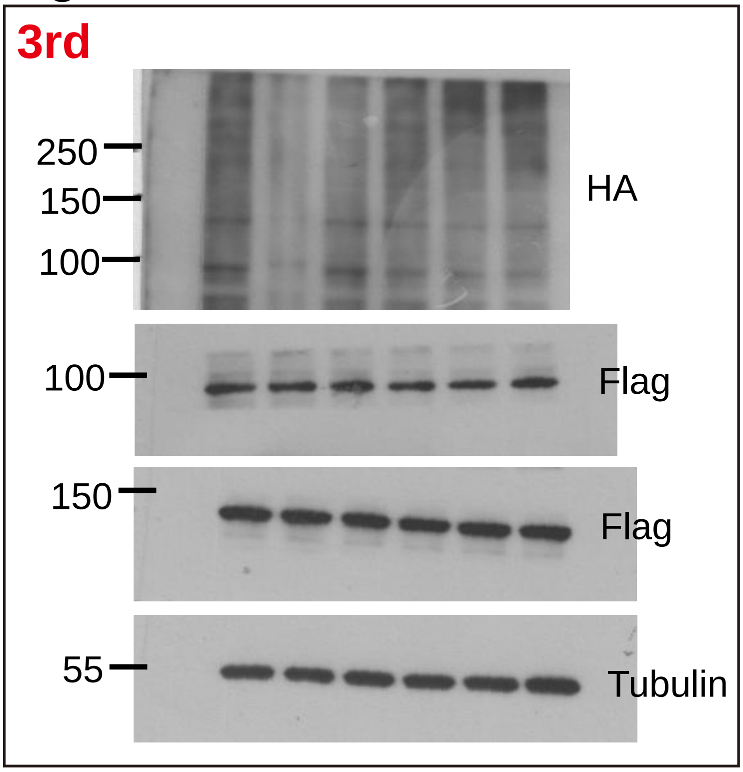

Fig.2f

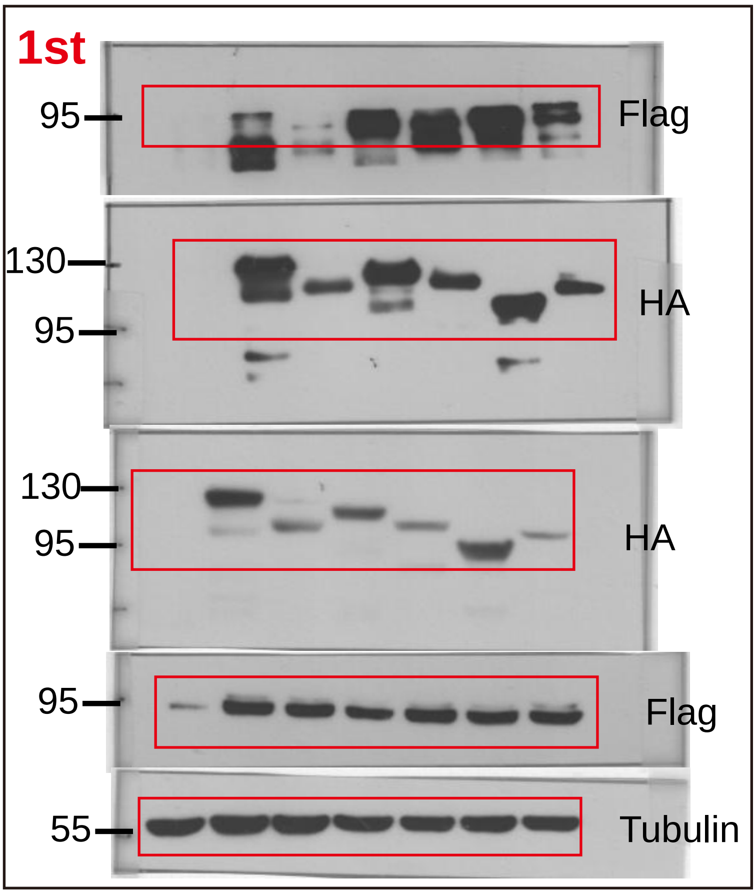

Fig.2f

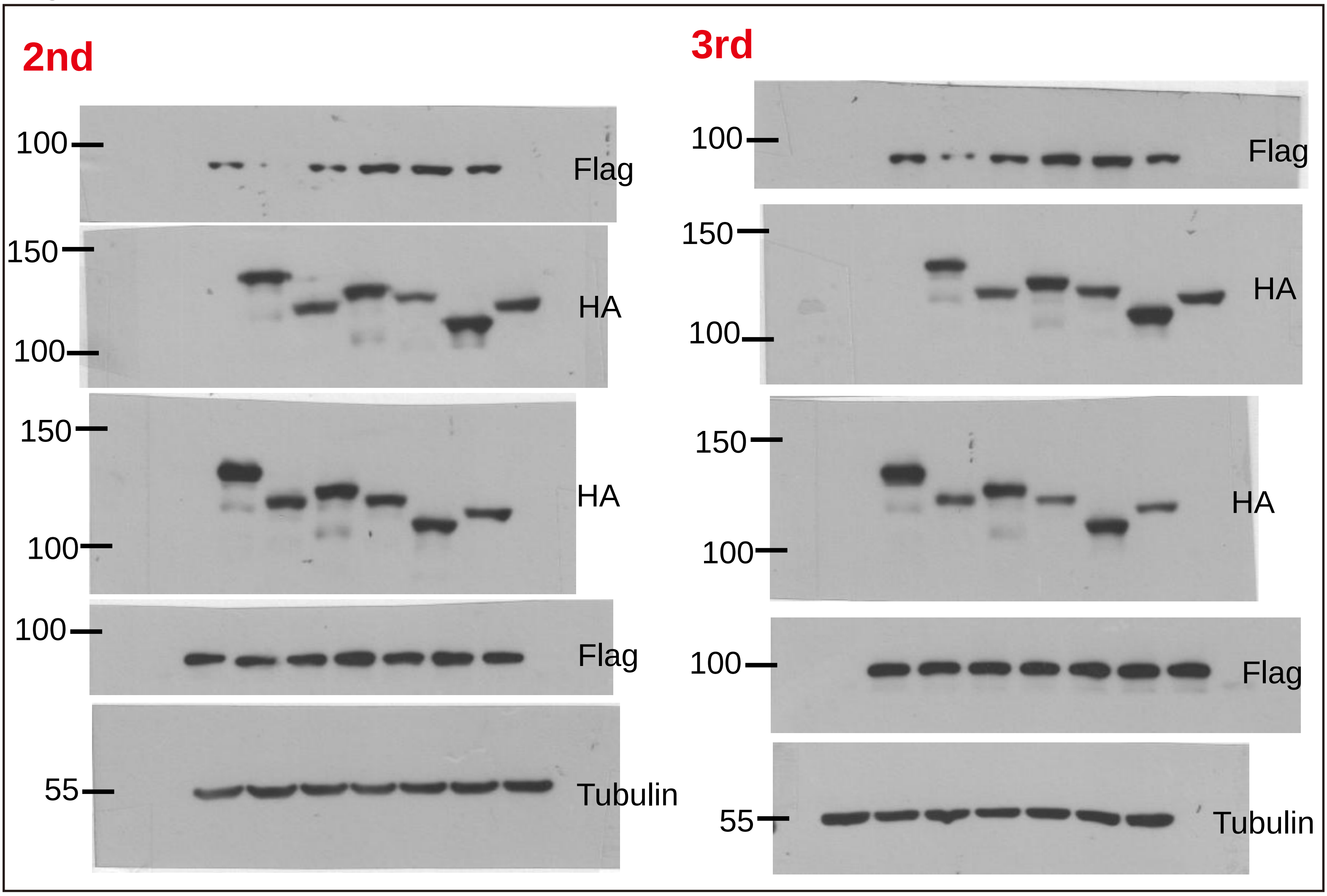

Fig.2g

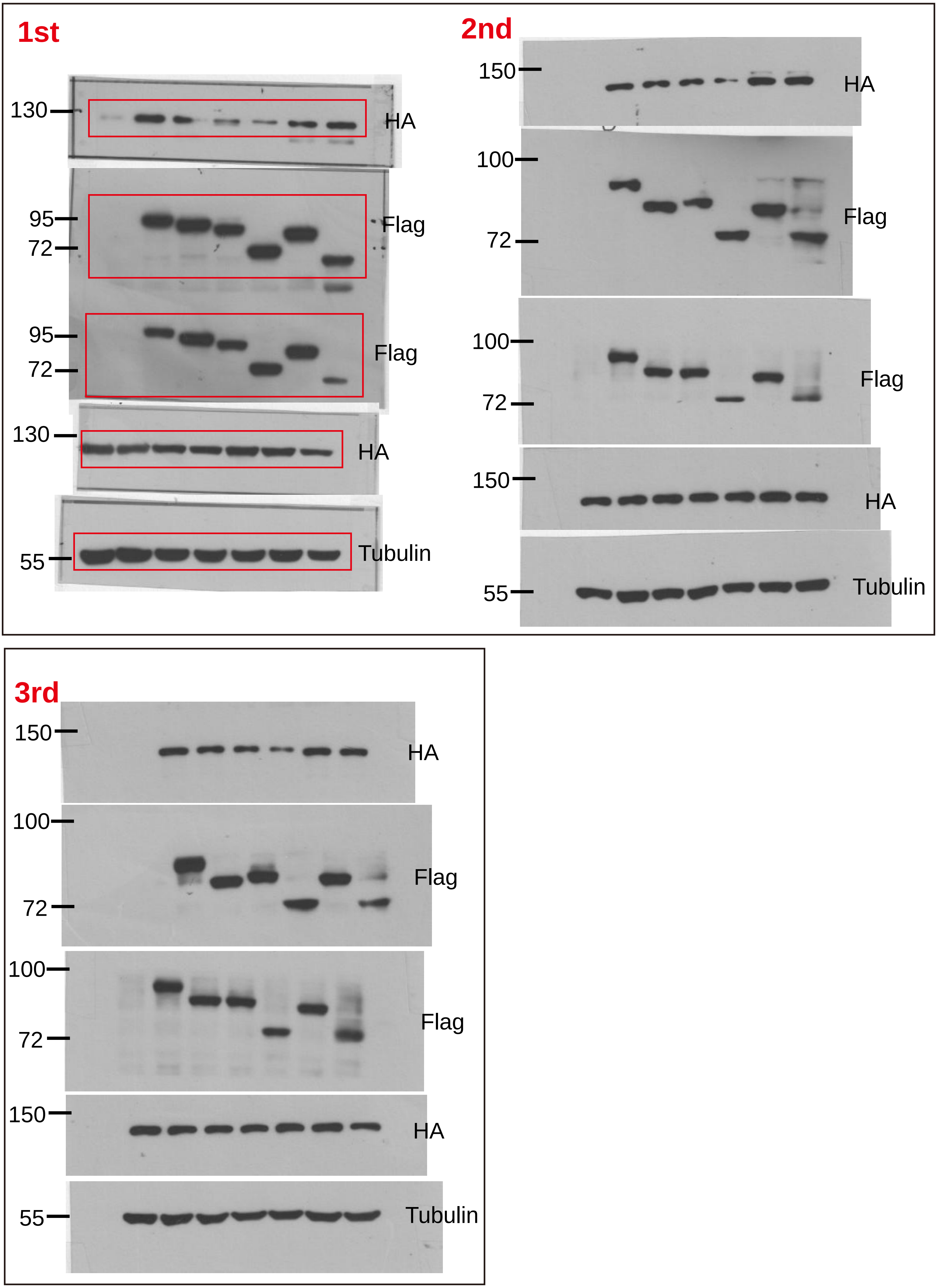
1st
130
HA
95
72
Flag
95
72
Flag
130
HA
55
Tubulin
2nd
150
HA
100
72
Flag
100
72
Flag
150
HA
55
Tubulin
3rd
150
HA
100
72
Flag
100
72
Flag
150
HA
55
Tubulin

Fig.2h

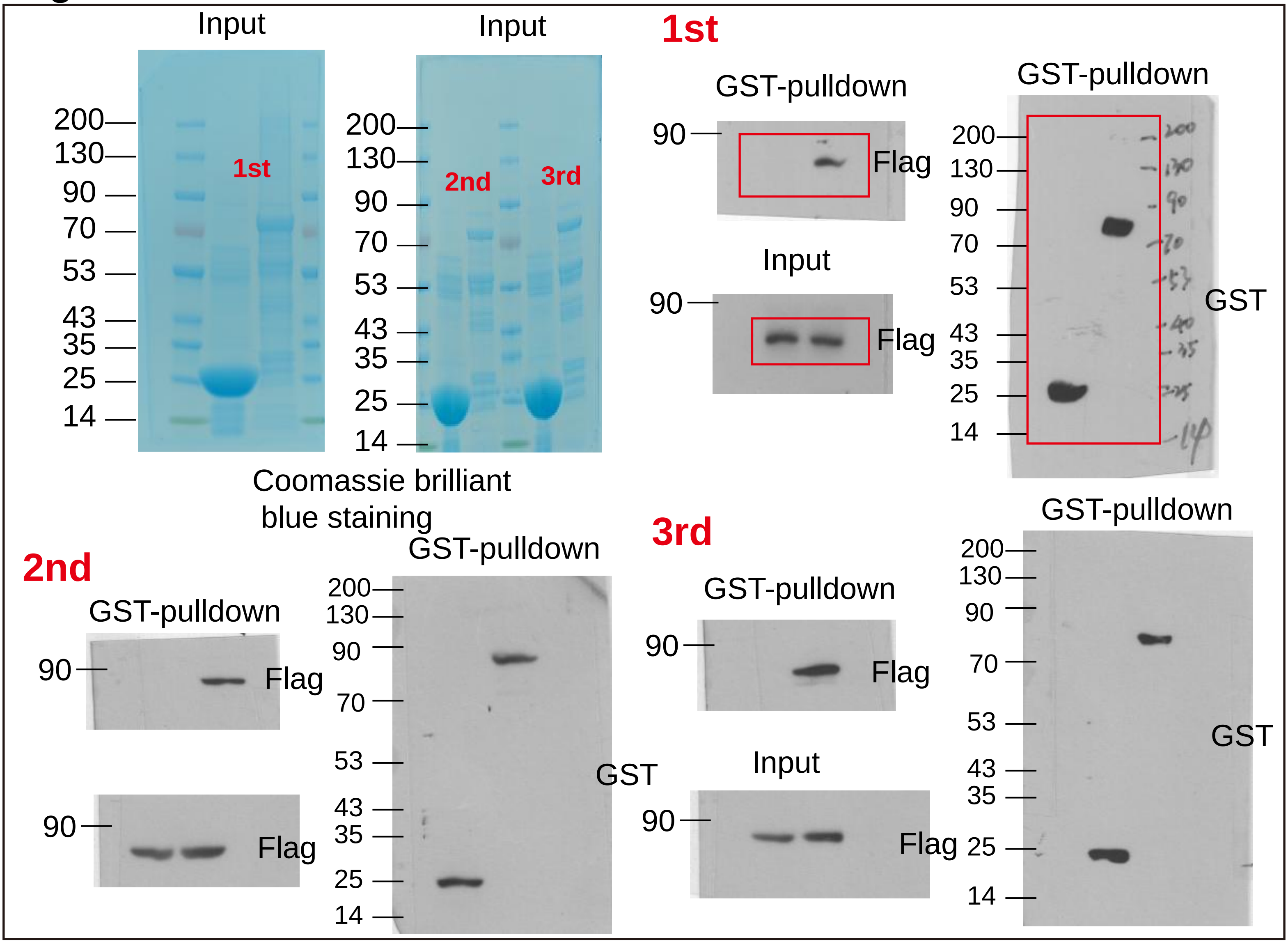
Input
Input
1st
2nd
3rd
200
130
90
70
53
43
35
25
14
Coomassie brilliant blue staining
GST-pulldown
Flag
Input
Flag
GST
2nd
GST-pulldown
Flag
Flag
GST-pulldown
GST
3rd
GST-pulldown
Flag
Input
Flag
GST-pulldown
GST

Fig.2i

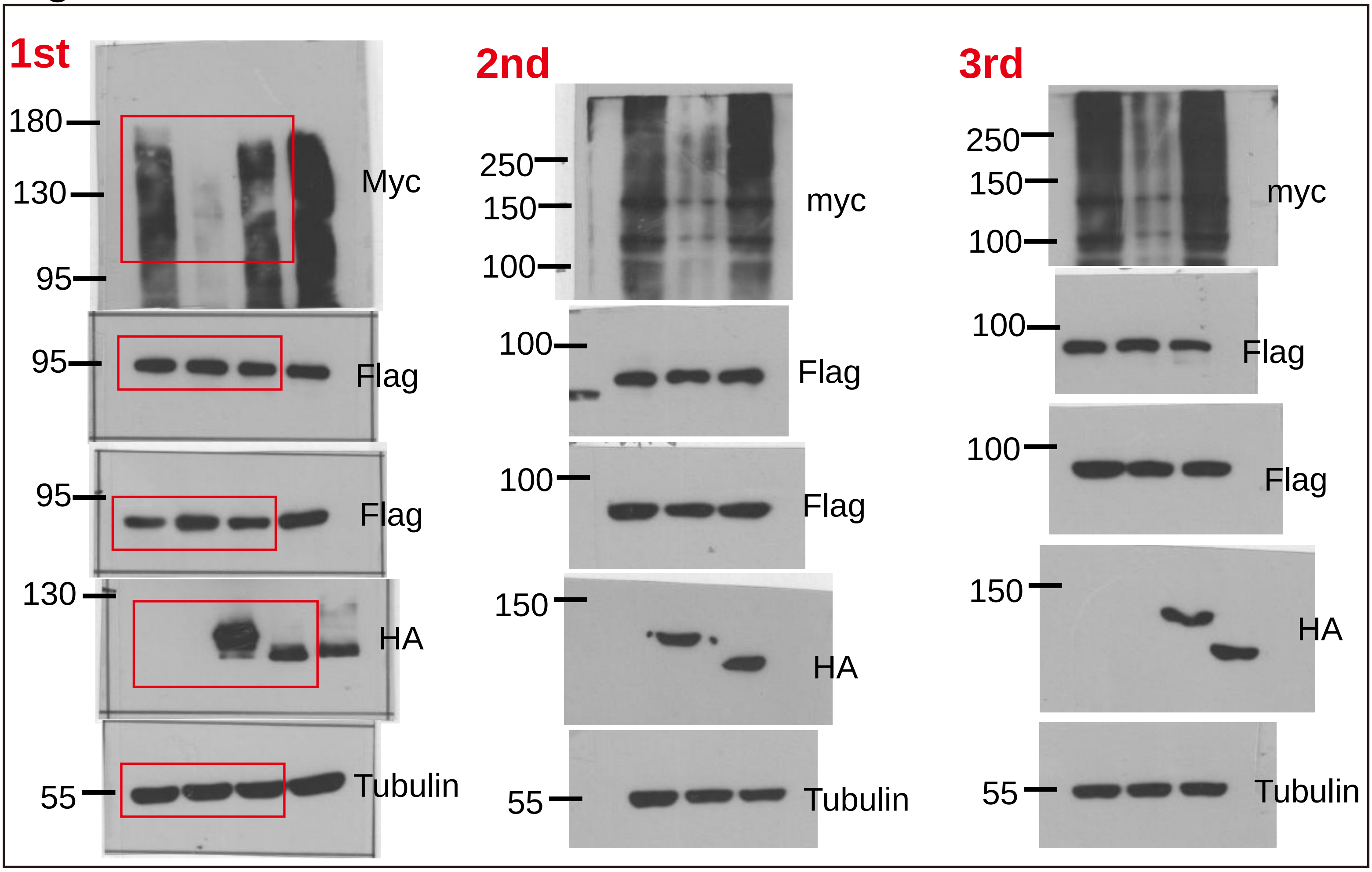
1st
180
130
95
Myc
Flag
Flag
HA
55
Tubulin
2nd
250
150
100
myc
Flag
Flag
HA
Tubulin
3rd
myc
Flag
Flag
HA
Tubulin

Fig.3f

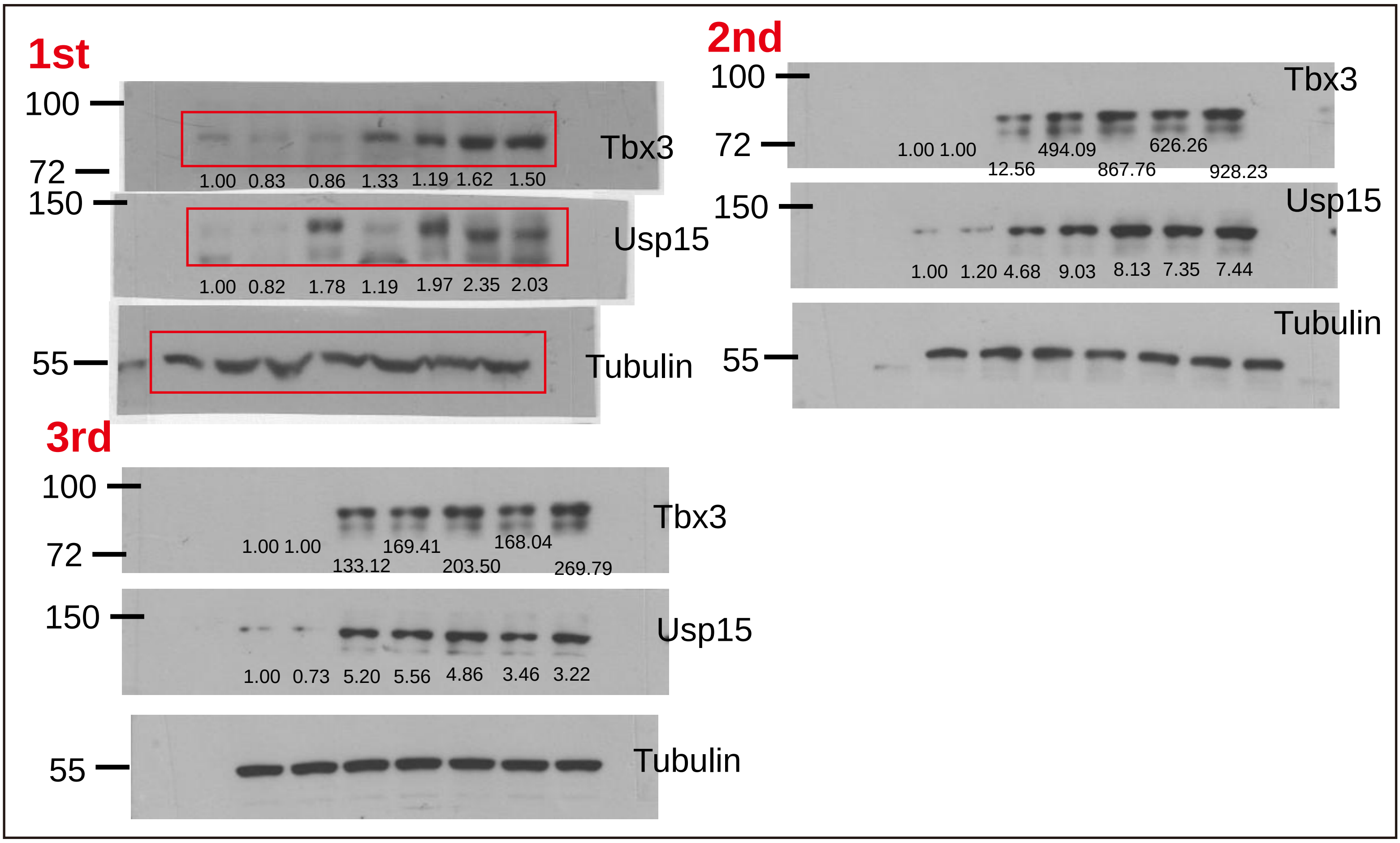
1st
100
72
Tbx3
1.00 0.83 0.86 1.33 1.19 1.62 1.50
150
Usp15
1.00 0.82 1.78 1.19 1.97 2.35 2.03
55
Tubulin
2nd
100
72
Tbx3
1.00 1.00 12.56 494.09 867.76 626.26 928.23
150
Usp15
1.00 1.20 4.68 9.03 8.13 7.35 7.44
55
Tubulin
3rd
100
72
Tbx3
1.00 1.00 133.12 169.41 203.50 168.04 269.79
150
Usp15
1.00 0.73 5.20 5.56 4.86 3.46 3.22
55
Tubulin

Fig.4a

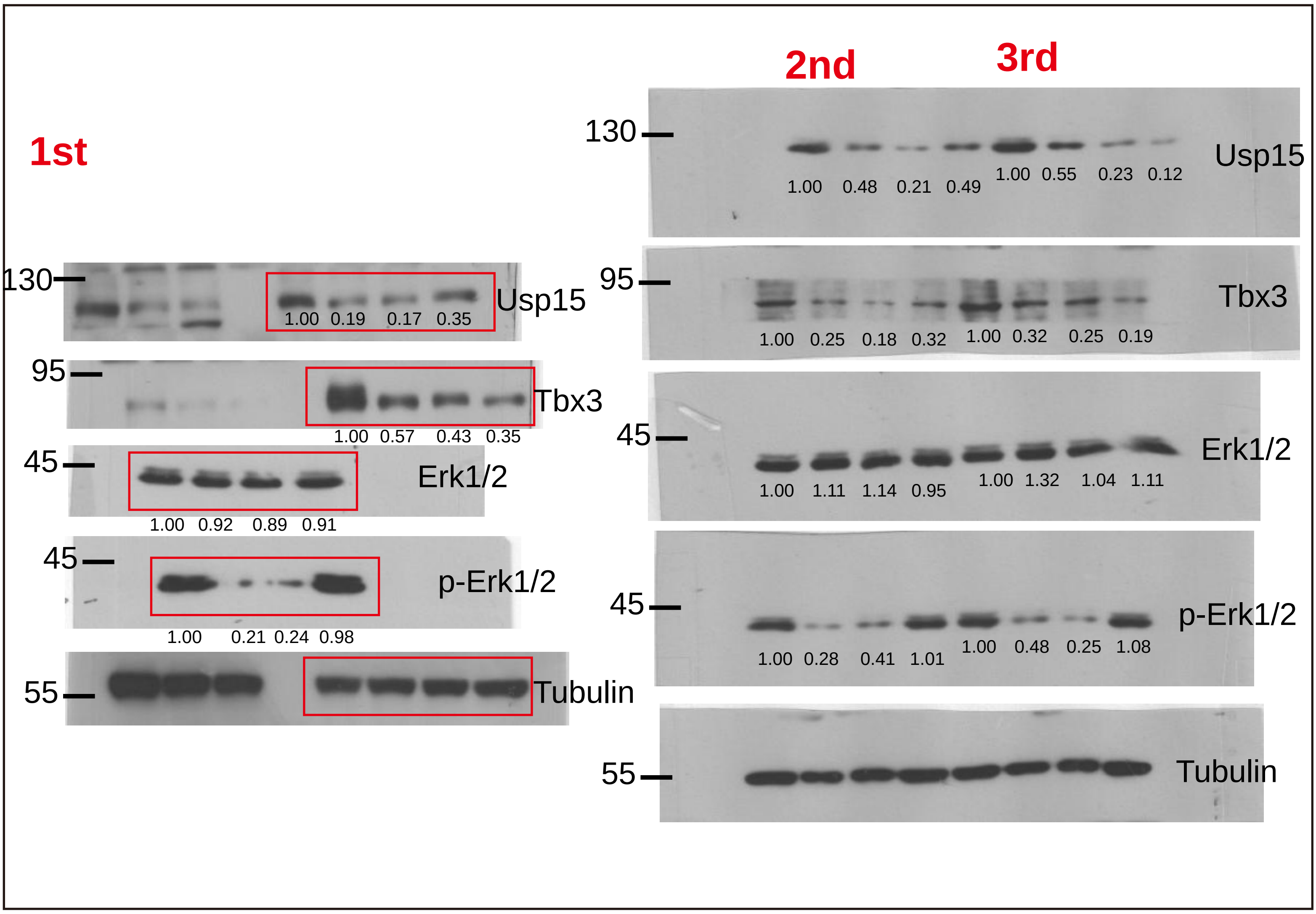
1st
130
Usp15
1.00 0.19 0.17 0.35
95
Tbx3
1.00 0.57 0.43 0.35
45
Erk1/2
1.00 0.92 0.89 0.91
45
p-Erk1/2
1.00 0.21 0.24 0.98
55
Tubulin
2nd
3rd
130
Usp15
1.00 0.48 0.21 0.49 1.00 0.55 0.23 0.12
95
Tbx3
1.00 0.25 0.18 0.32 1.00 0.32 0.25 0.19
45
Erk1/2
1.00 1.11 1.14 0.95 1.00 1.32 1.04 1.11
45
p-Erk1/2
1.00 0.28 0.41 1.01 1.00 0.48 0.25 1.08
55
Tubulin

Fig.4b

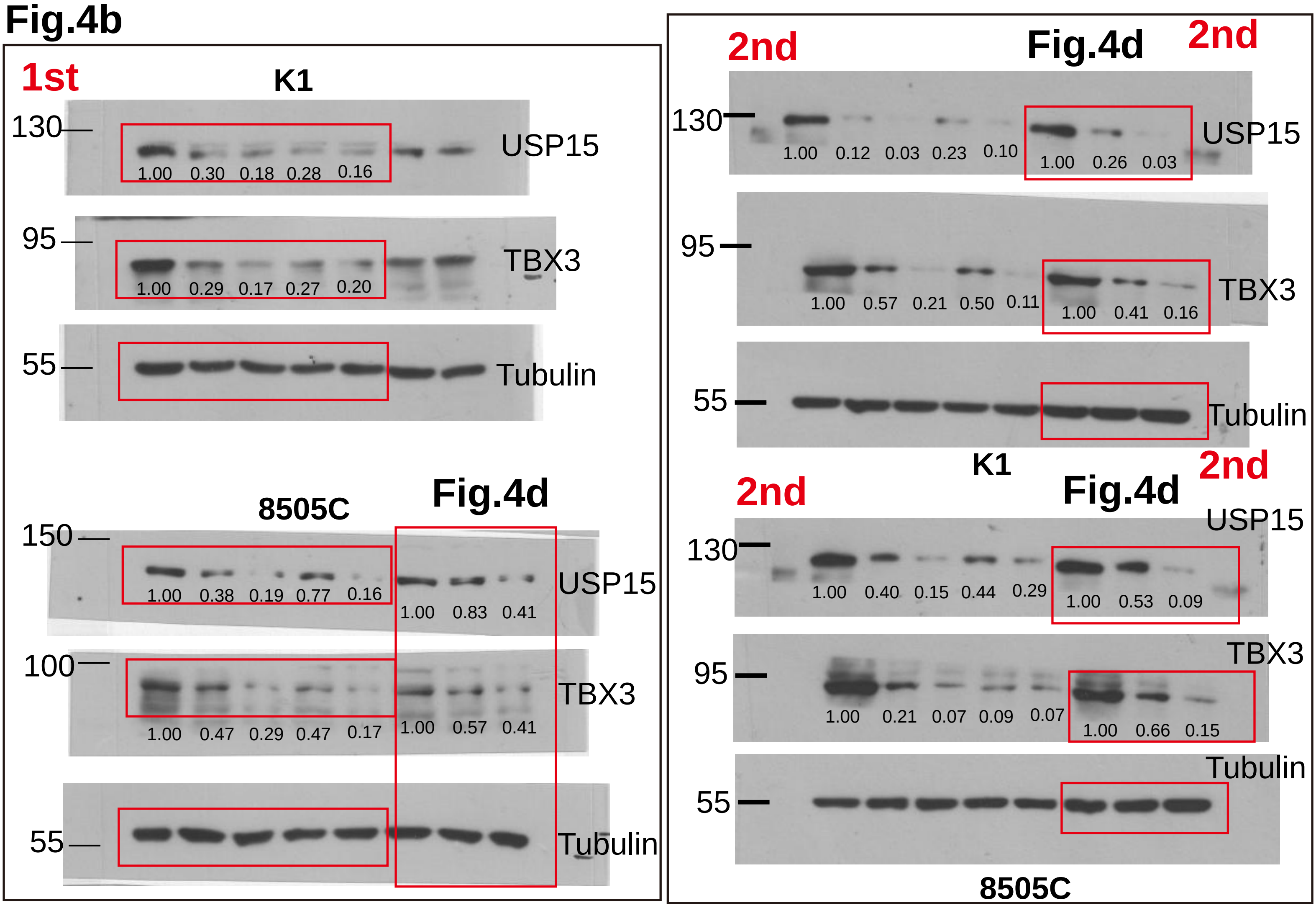
1st
K1
130
USP15
1.00 0.30 0.18 0.28 0.16
95
TBX3
1.00 0.29 0.17 0.27 0.20
55
Tubulin
8505C
Fig.4d
150
USP15
1.00 0.38 0.19 0.77 0.16
1.00 0.83 0.41
100
TBX3
1.00 0.47 0.29 0.47 0.17
1.00 0.57 0.41
55
Tubulin
2nd
Fig.4d
2nd
130
USP15
1.00 0.12 0.03 0.23 0.10
1.00 0.26 0.03
95
TBX3
1.00 0.57 0.21 0.50 0.11
1.00 0.41 0.16
55
Tubulin
K1
2nd
Fig.4d
2nd
130
USP15
1.00 0.40 0.15 0.44 0.29
1.00 0.53 0.09
95
TBX3
1.00 0.21 0.07 0.09 0.07
1.00 0.66 0.15
55
Tubulin
8505C

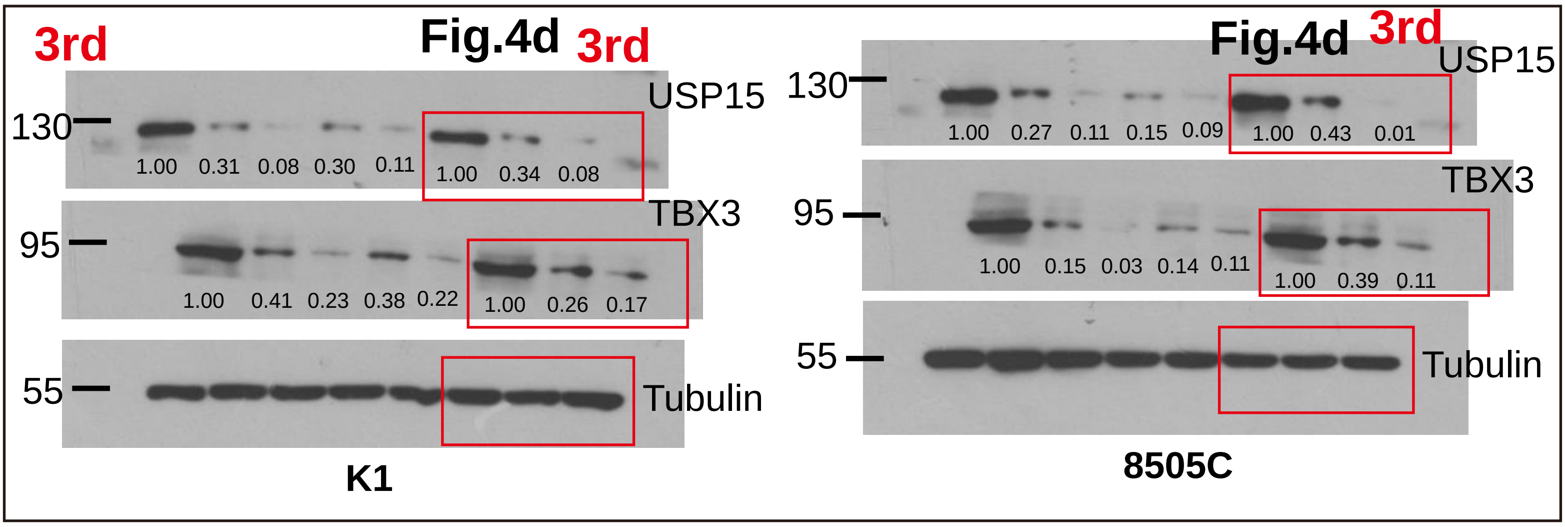
3rd
Fig.4d
3rd
130
USP15
1.00 0.31 0.08 0.30 0.11
1.00 0.34 0.08
95
TBX3
1.00 0.41 0.23 0.38 0.22
1.00 0.26 0.17
55
Tubulin
K1
Fig.4d
3rd
130
USP15
1.00 0.27 0.11 0.15 0.09
1.00 0.43 0.01
95
TBX3
1.00 0.15 0.03 0.14 0.11
1.00 0.39 0.11
55
Tubulin
8505C

Fig.4c

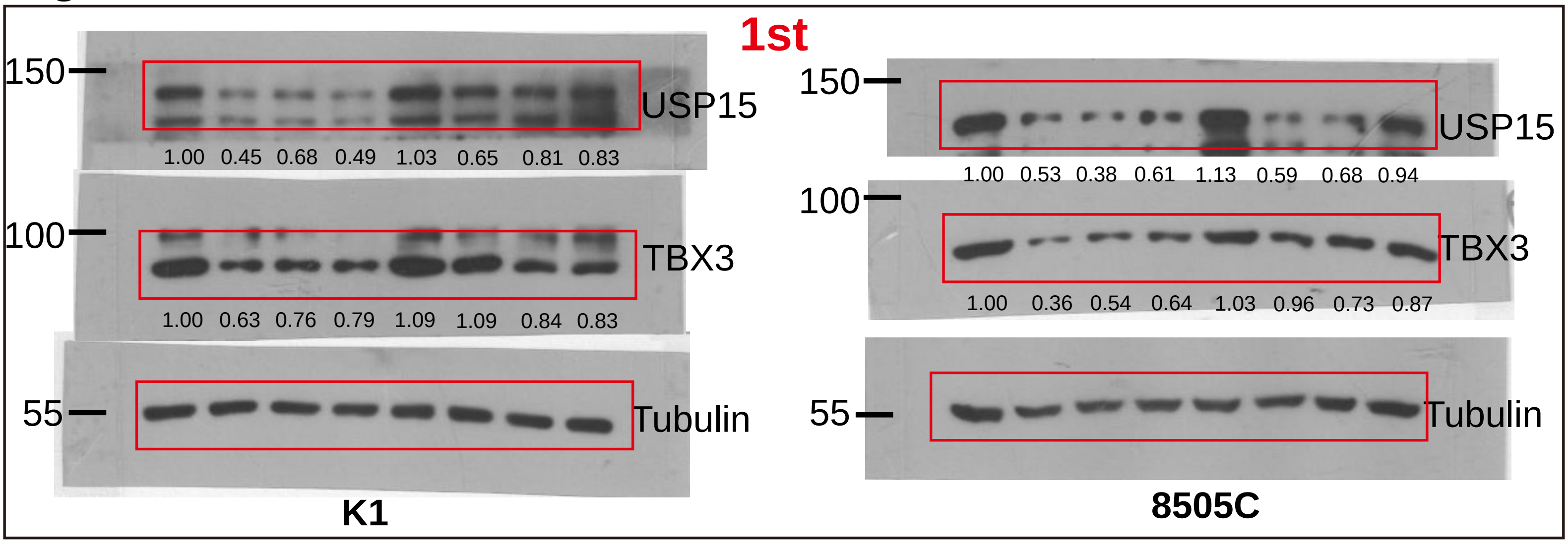
1st
150
USP15
1.00 0.45 0.68 0.49 1.03 0.65 0.81 0.83
100
TBX3
1.00 0.63 0.76 0.79 1.09 1.09 0.84 0.83
55
Tubulin
K1
150
USP15
1.00 0.53 0.38 0.61 1.13 0.59 0.68 0.94
100
TBX3
1.00 0.36 0.54 0.64 1.03 0.96 0.73 0.87
55
Tubulin
8505C

Fig.4c

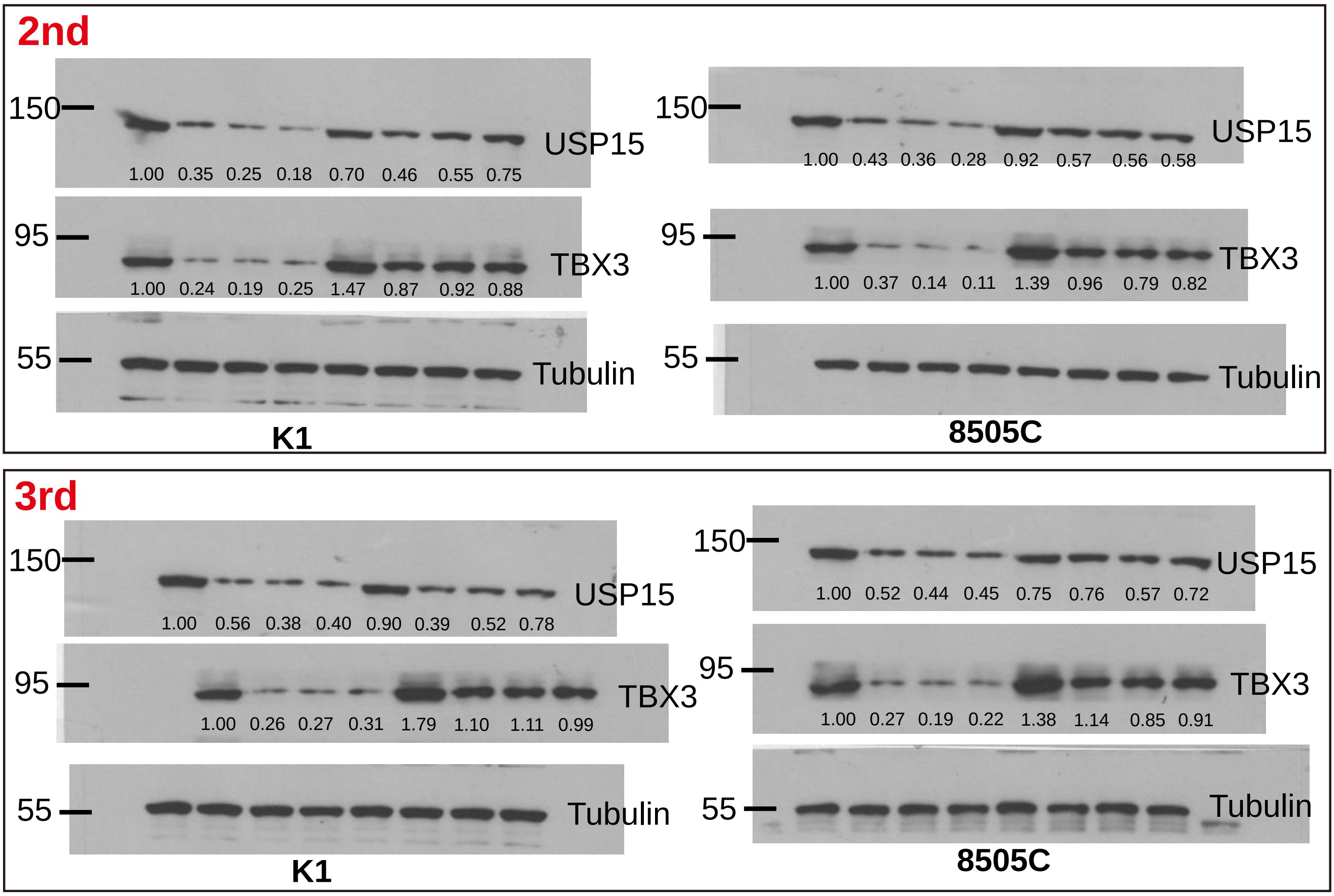

Fig.4d

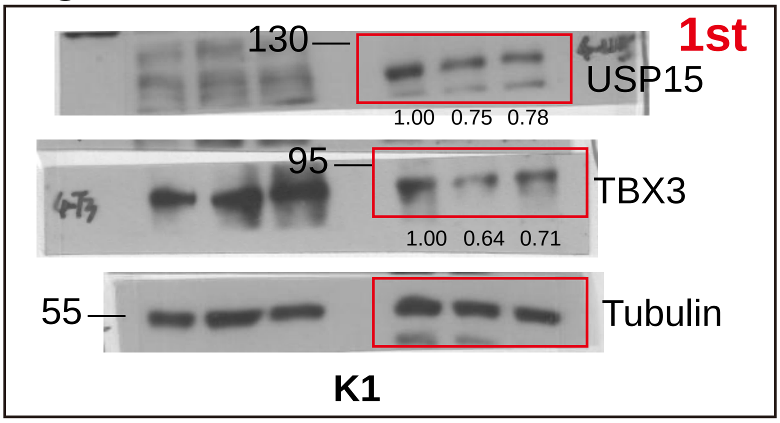

Fig.4g

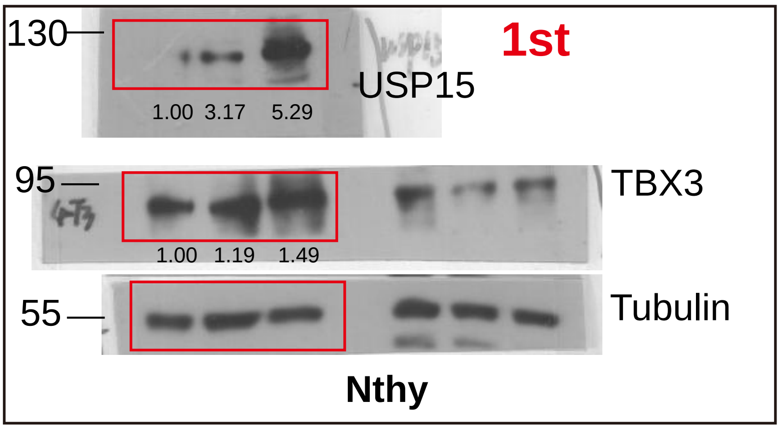

Fig.4g

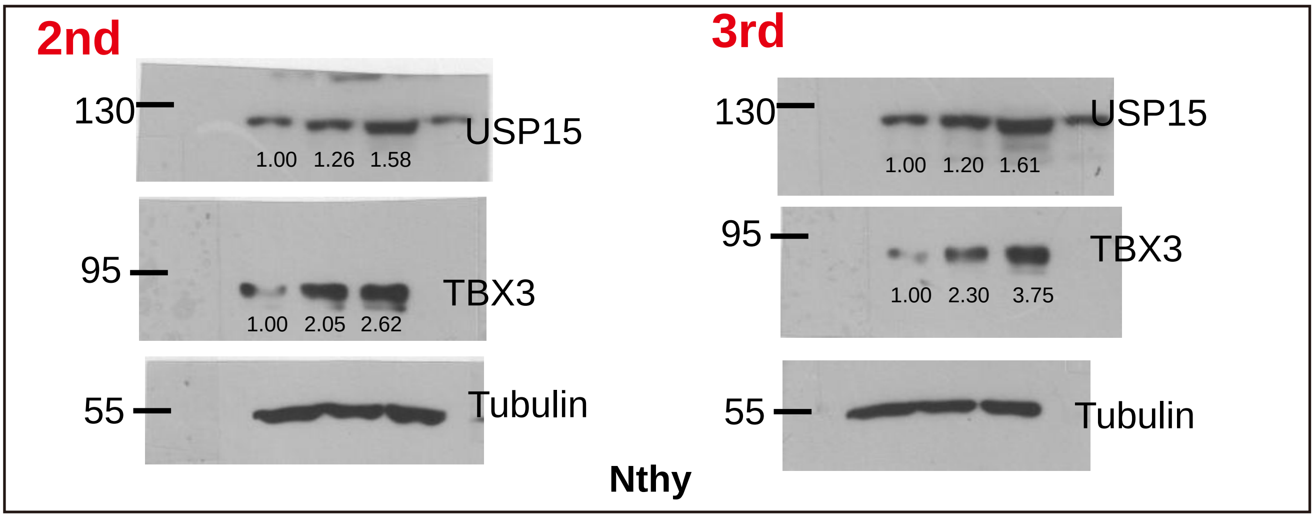

Fig.5i, j

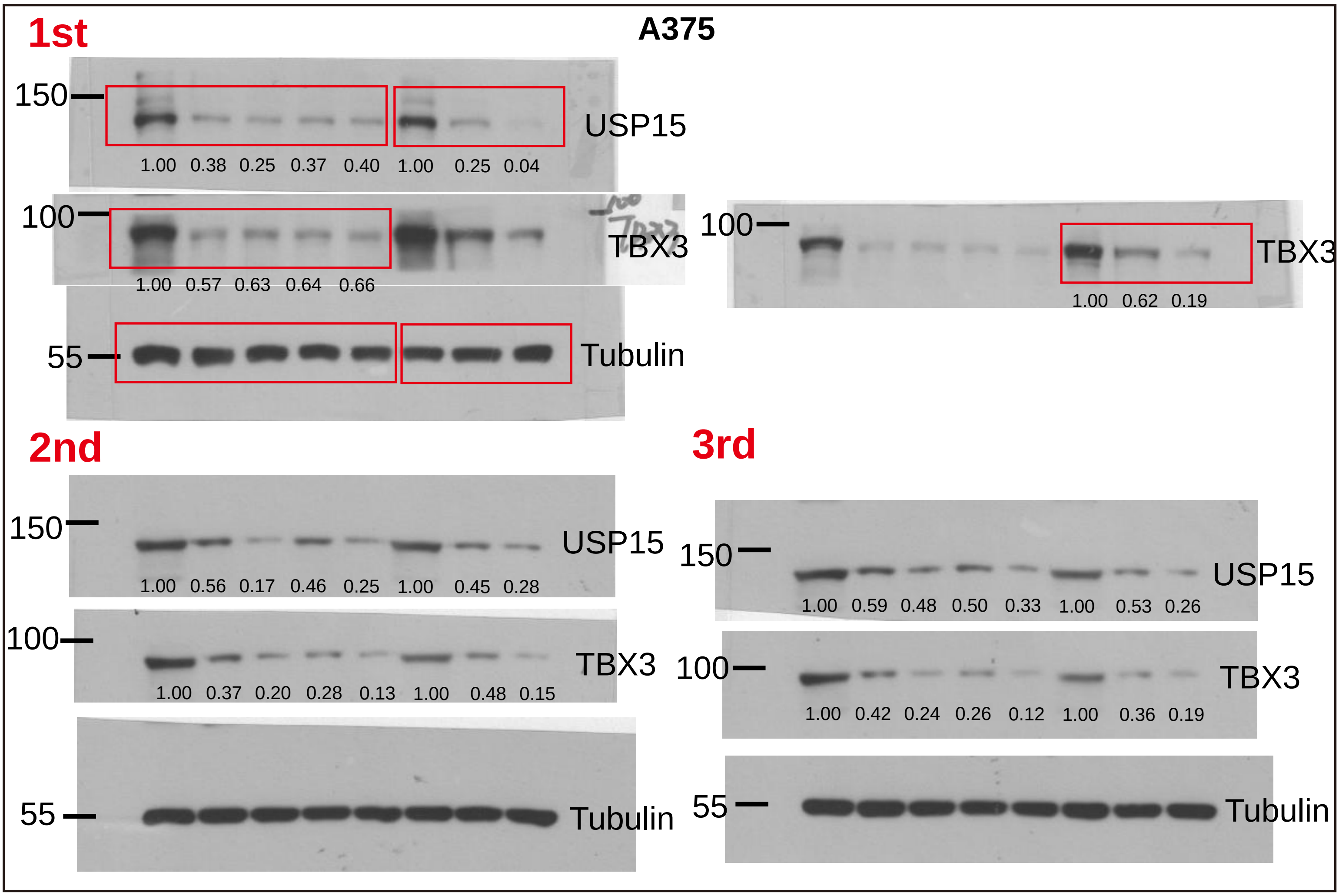

Fig.6b

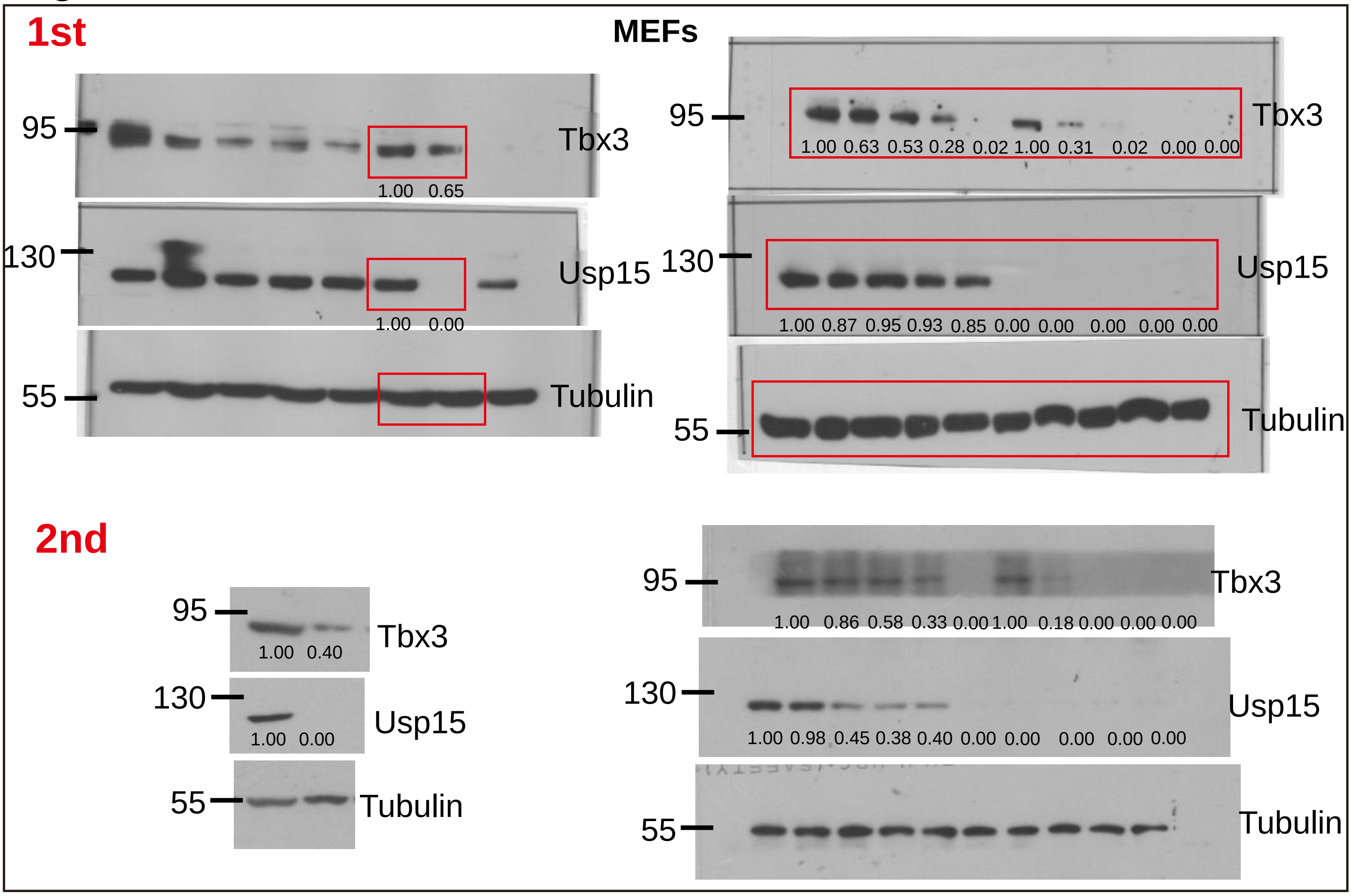

Fig.6b

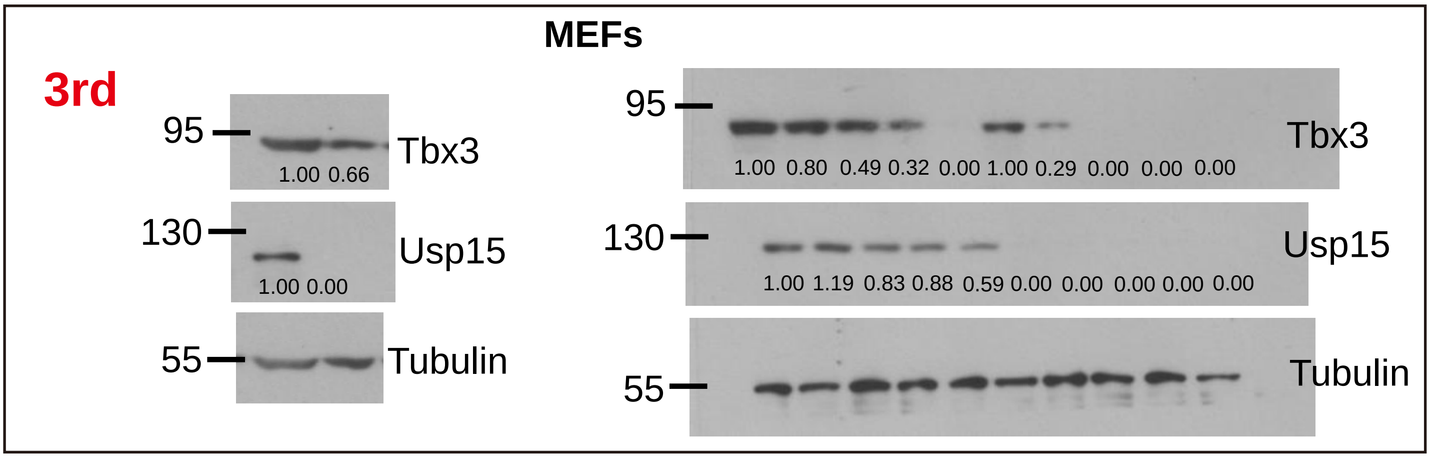
MEFs
3rd
95
130
55
Tbx3
Usp15
Tubulin
1.00 0.66
1.00 0.00
1.00 0.80 0.49 0.32 0.00 1.00 0.29 0.00 0.00 0.00
1.00 1.19 0.83 0.88 0.59 0.00 0.00 0.00 0.00 0.00

Fig.6d

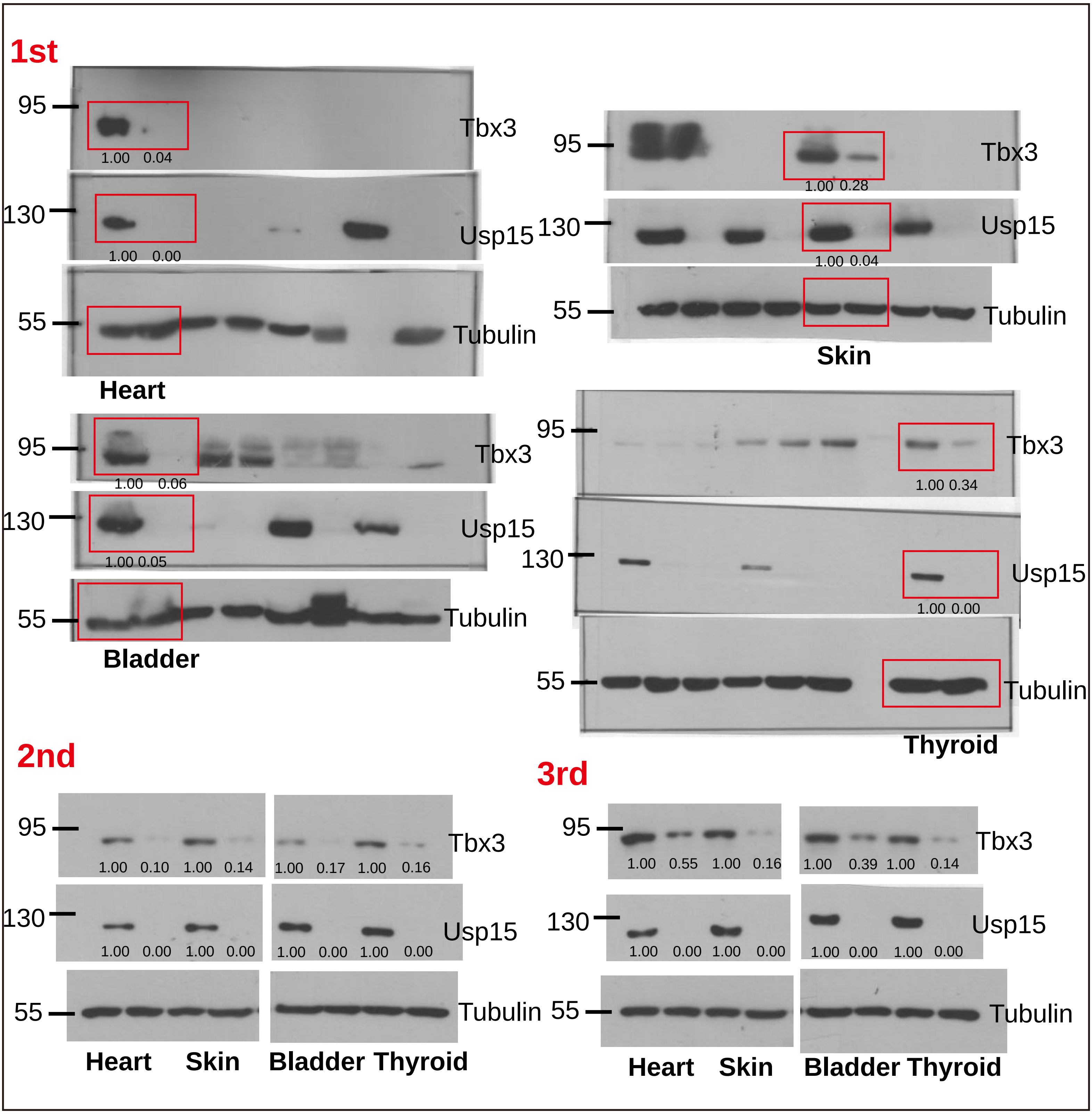
1st
95
130
55
Tbx3
Usp15
Tubulin
1.00 0.04
1.00 0.00
Heart
1.00 0.06
1.00 0.05
Bladder
1.00 0.28
1.00 0.04
Skin
1.00 0.34
1.00 0.00
Thyroid
2nd
1.00 0.10 1.00 0.14
1.00 0.17 1.00 0.16
1.00 0.00 1.00 0.00
1.00 0.00 1.00 0.00
Heart Skin Bladder Thyroid
3rd
1.00 0.55 1.00 0.16
1.00 0.39 1.00 0.14
1.00 0.00 1.00 0.00
1.00 0.00 1.00 0.00
Heart Skin Bladder Thyroid

Fig.7f

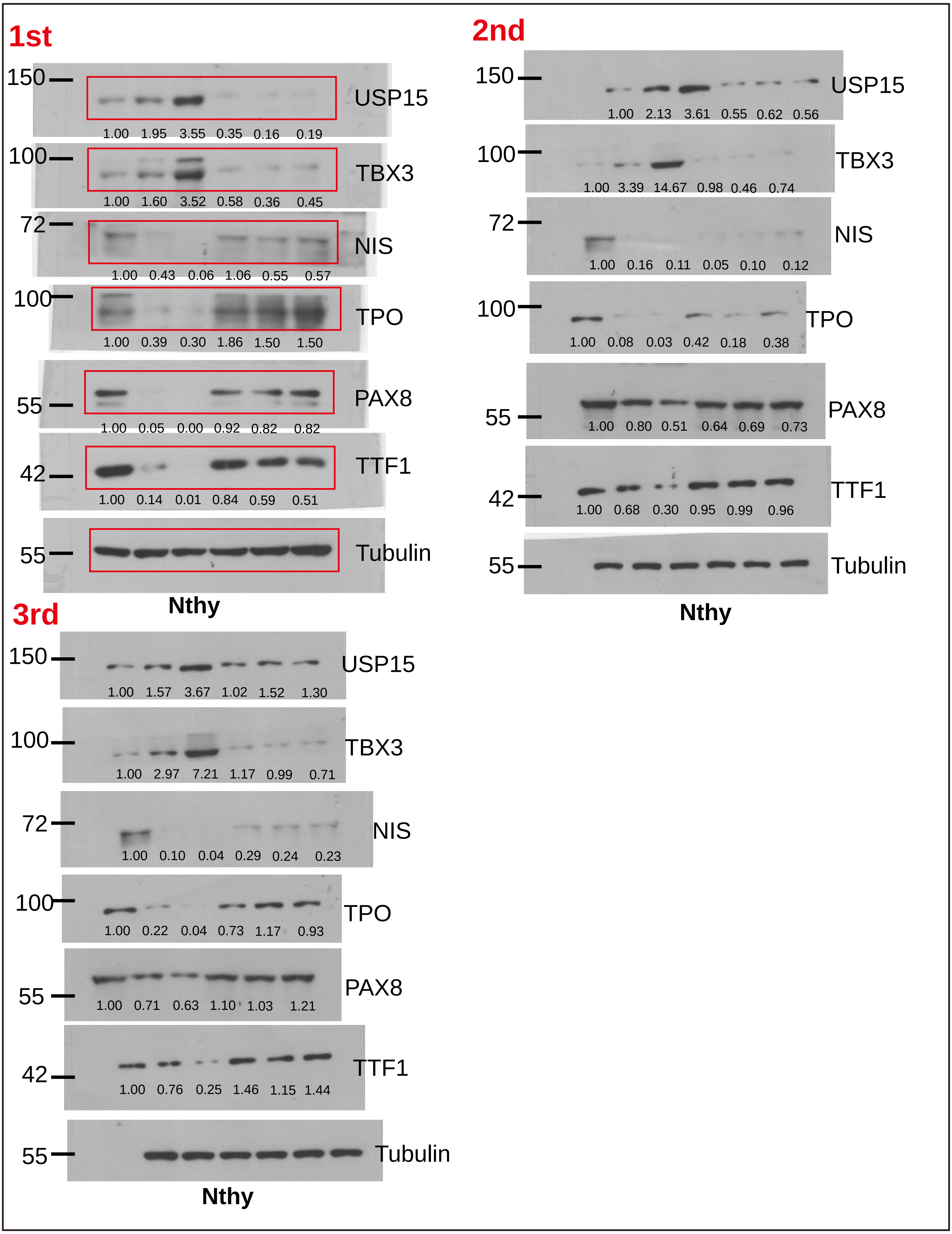
1st
150
USP15
1.00 1.95 3.55 0.35 0.16 0.19
100
TBX3
1.00 1.60 3.52 0.58 0.36 0.45
72
NIS
1.00 0.43 0.06 1.06 0.55 0.57
100
TPO
1.00 0.39 0.30 1.86 1.50 1.50
55
PAX8
1.00 0.05 0.00 0.92 0.82 0.82
42
TTF1
1.00 0.14 0.01 0.84 0.59 0.51
55
Tubulin
Nthy
2nd
150
USP15
1.00 2.13 3.61 0.55 0.62 0.56
100
TBX3
1.00 3.39 14.67 0.98 0.46 0.74
72
NIS
1.00 0.16 0.11 0.05 0.10 0.12
100
TPO
1.00 0.08 0.03 0.42 0.18 0.38
55
PAX8
1.00 0.80 0.51 0.64 0.69 0.73
42
TTF1
1.00 0.68 0.30 0.95 0.99 0.96
55
Tubulin
Nthy
3rd
150
USP15
1.00 1.57 3.67 1.02 1.52 1.30
100
TBX3
1.00 2.97 7.21 1.17 0.99 0.71
72
NIS
1.00 0.10 0.04 0.29 0.24 0.23
100
TPO
1.00 0.22 0.04 0.73 1.17 0.93
55
PAX8
1.00 0.71 0.63 1.10 1.03 1.21
42
TTF1
1.00 0.76 0.25 1.46 1.15 1.44
55
Tubulin
Nthy

Supplementary Fig.1a

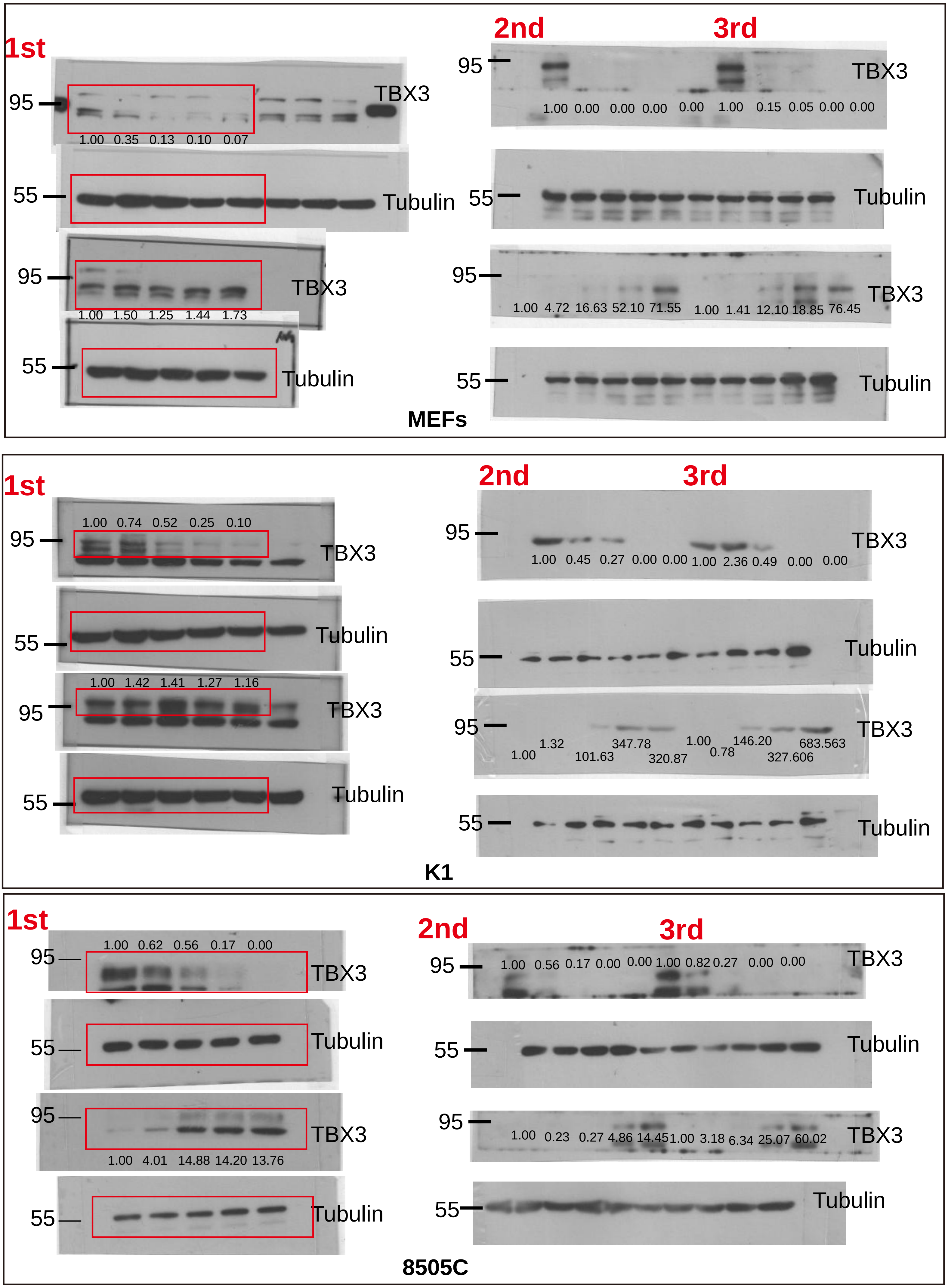

Supplementary Fig.1a

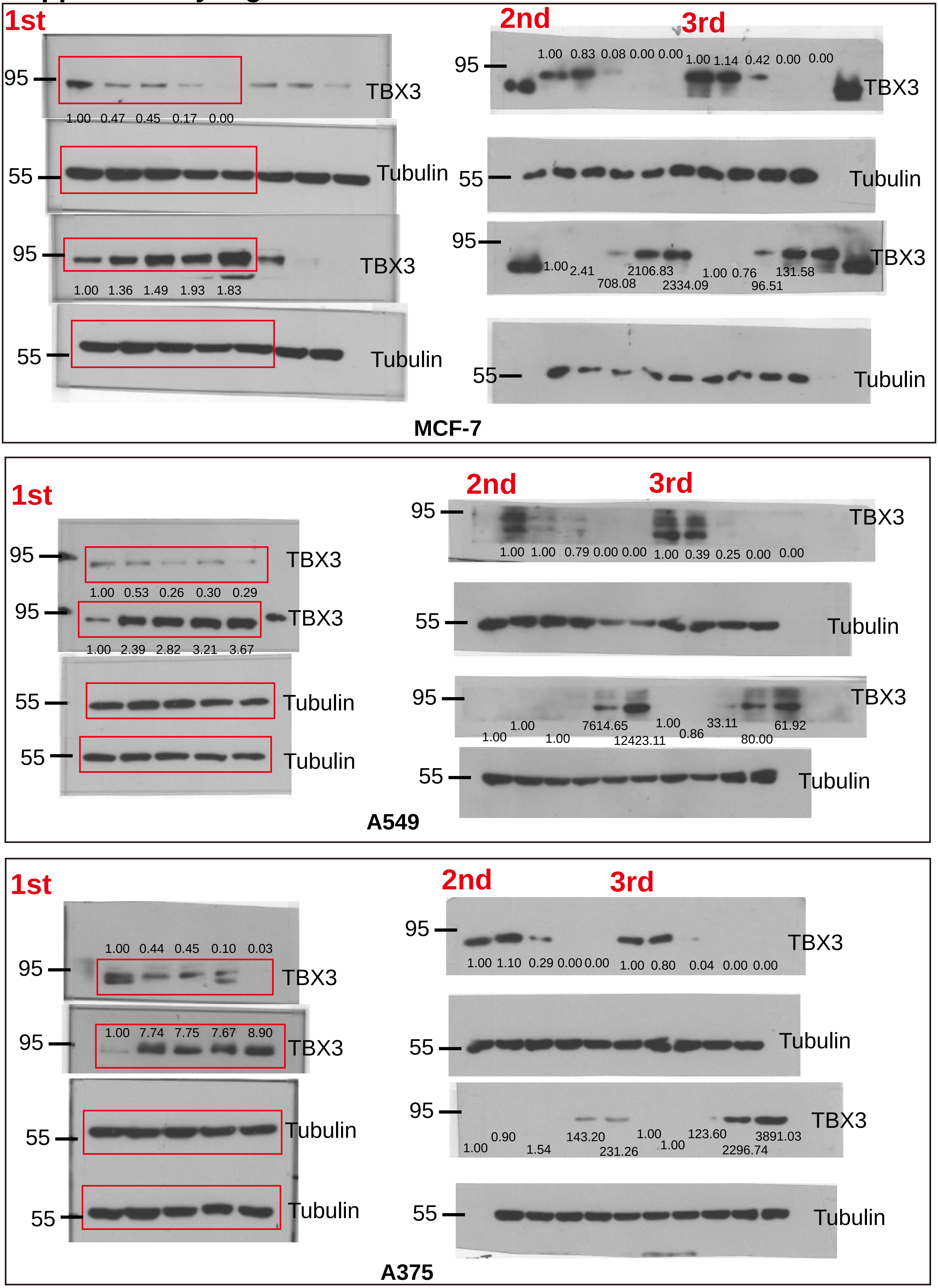

Supplementary Fig.1b

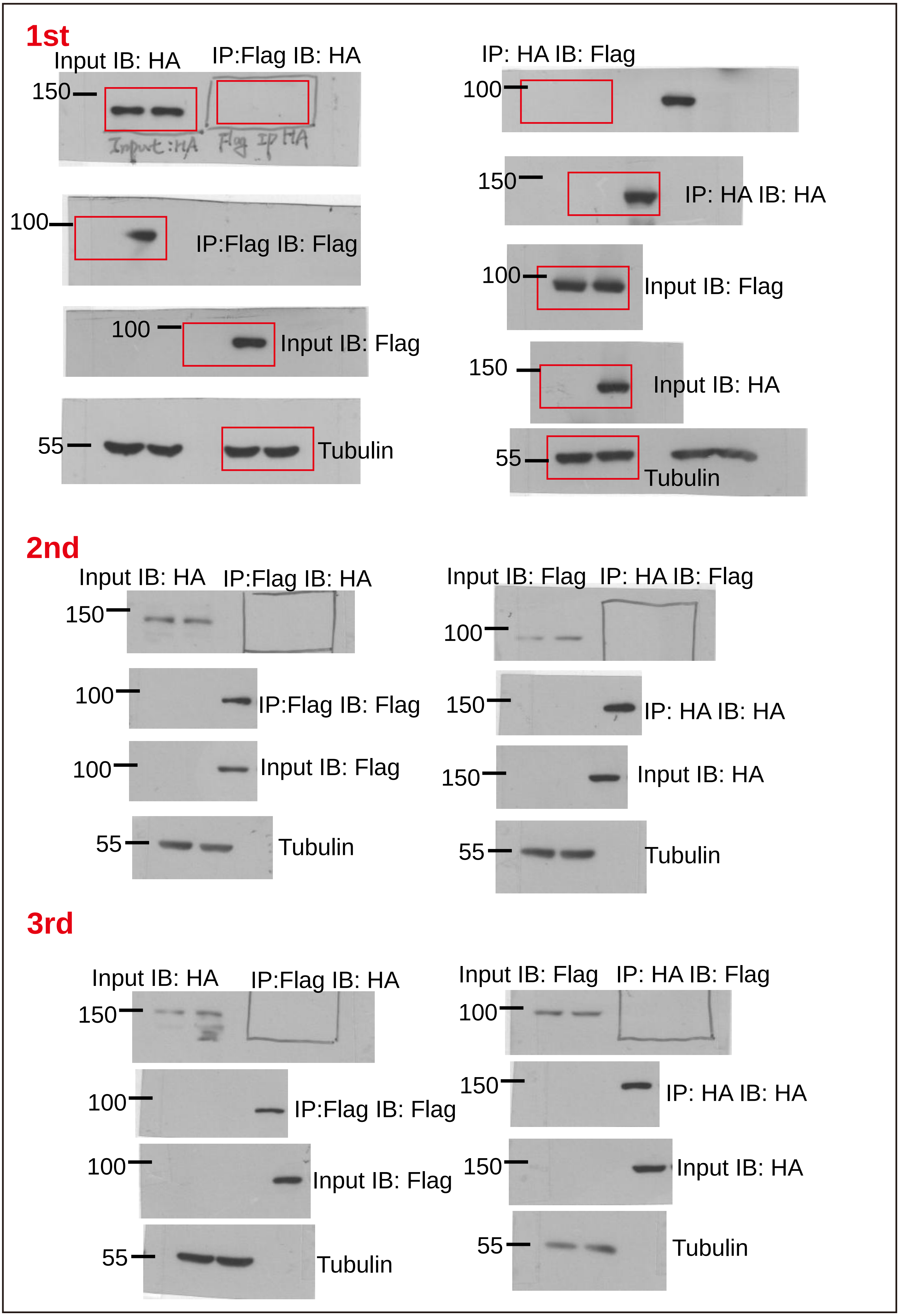

Supplementary Fig.1e

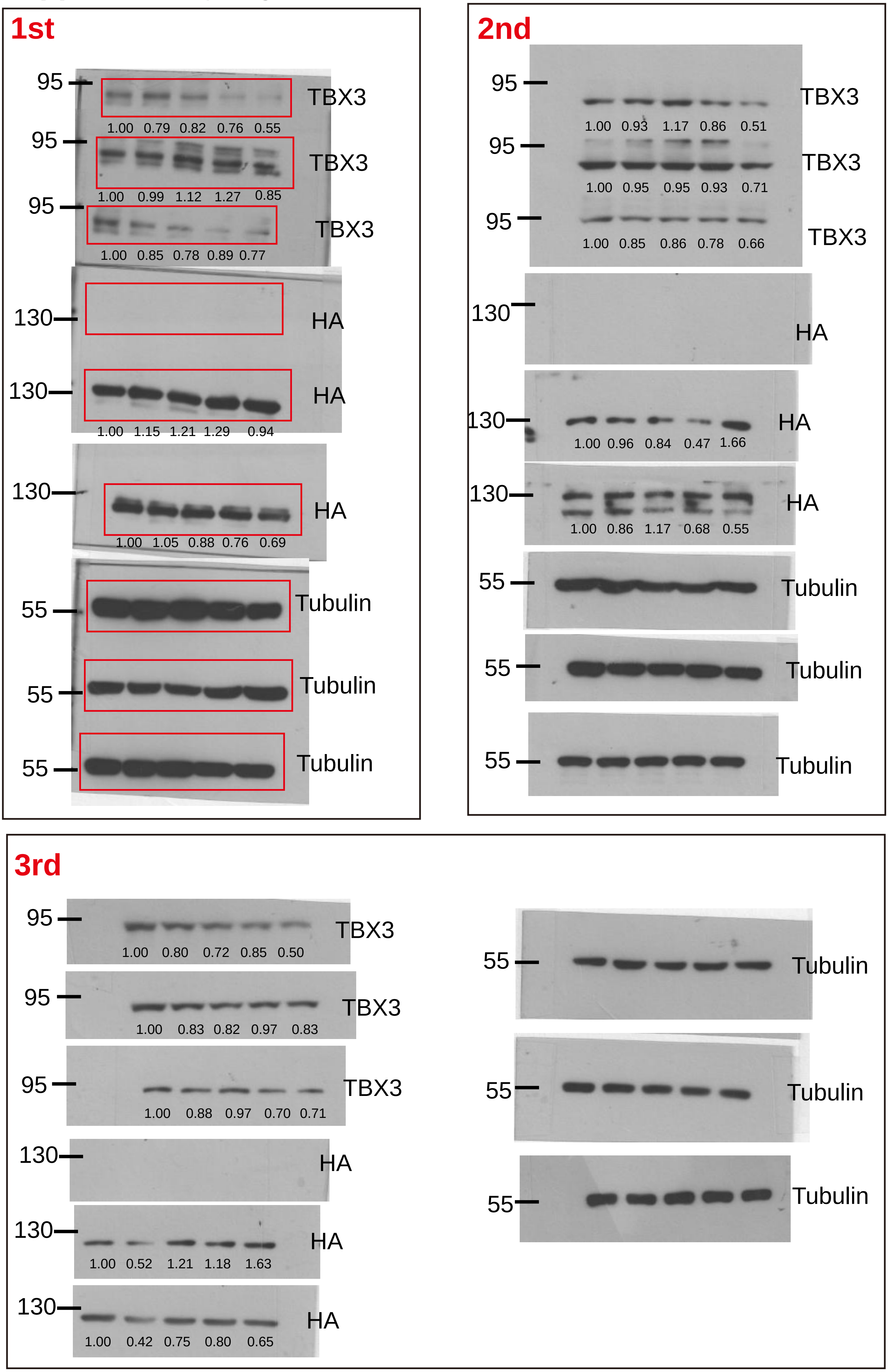

Supplementary Fig.1f

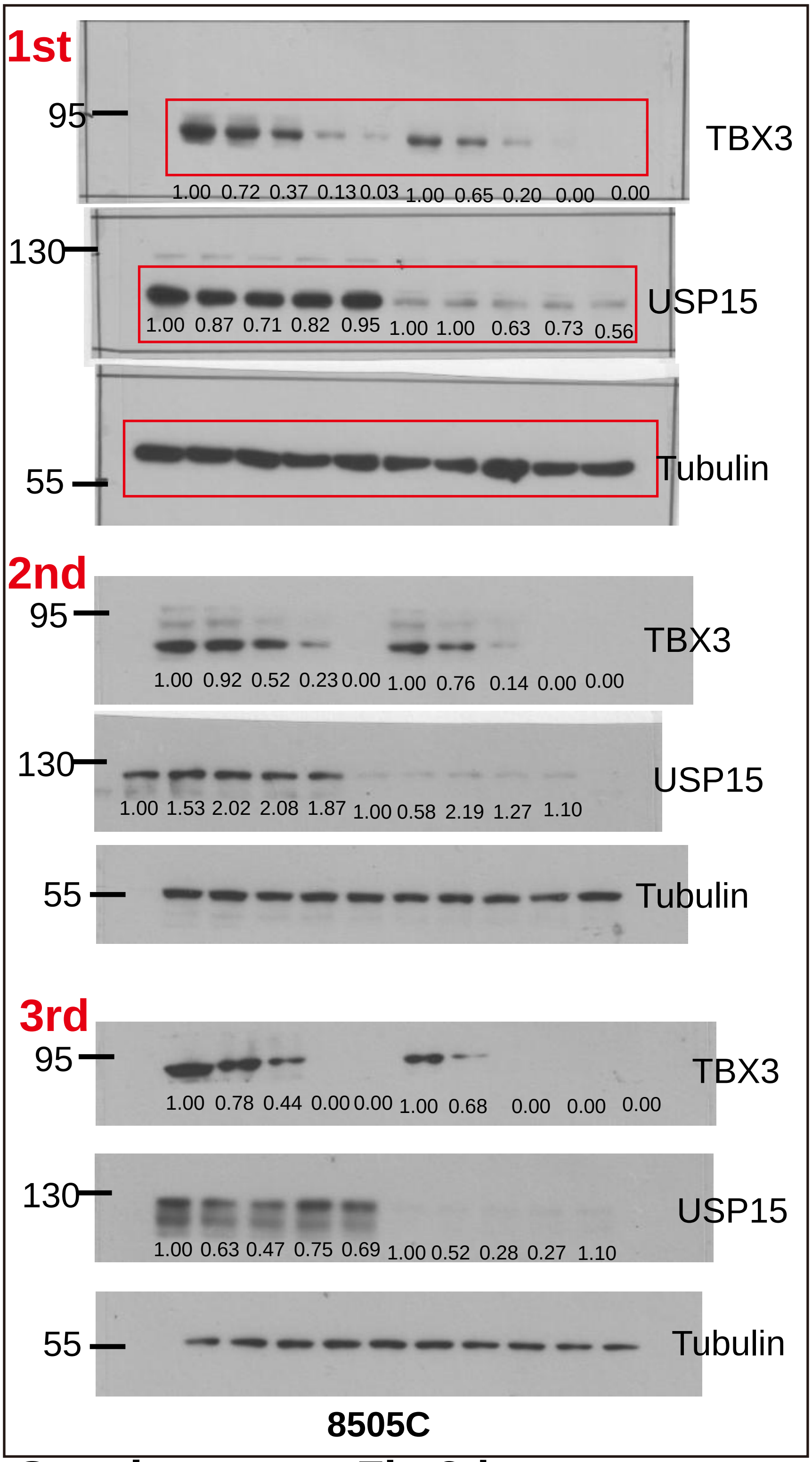

Supplementary Fig.2a

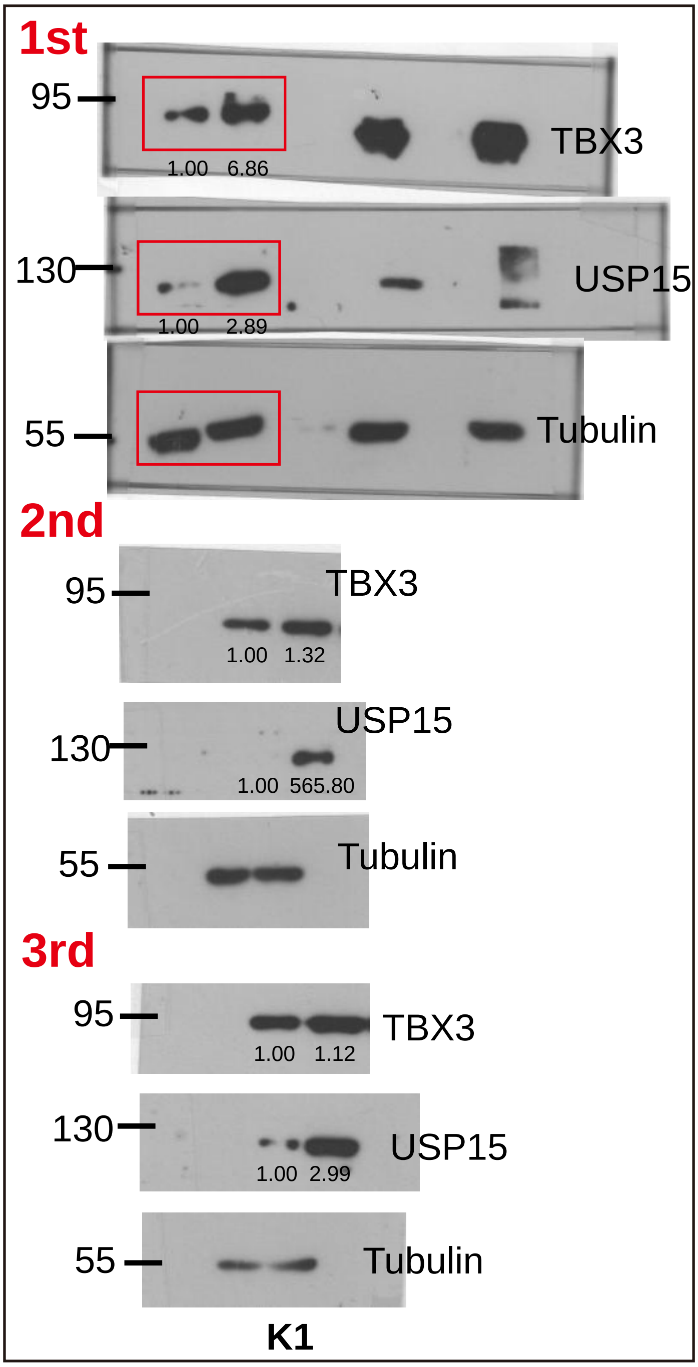

Supplementary Fig.2d

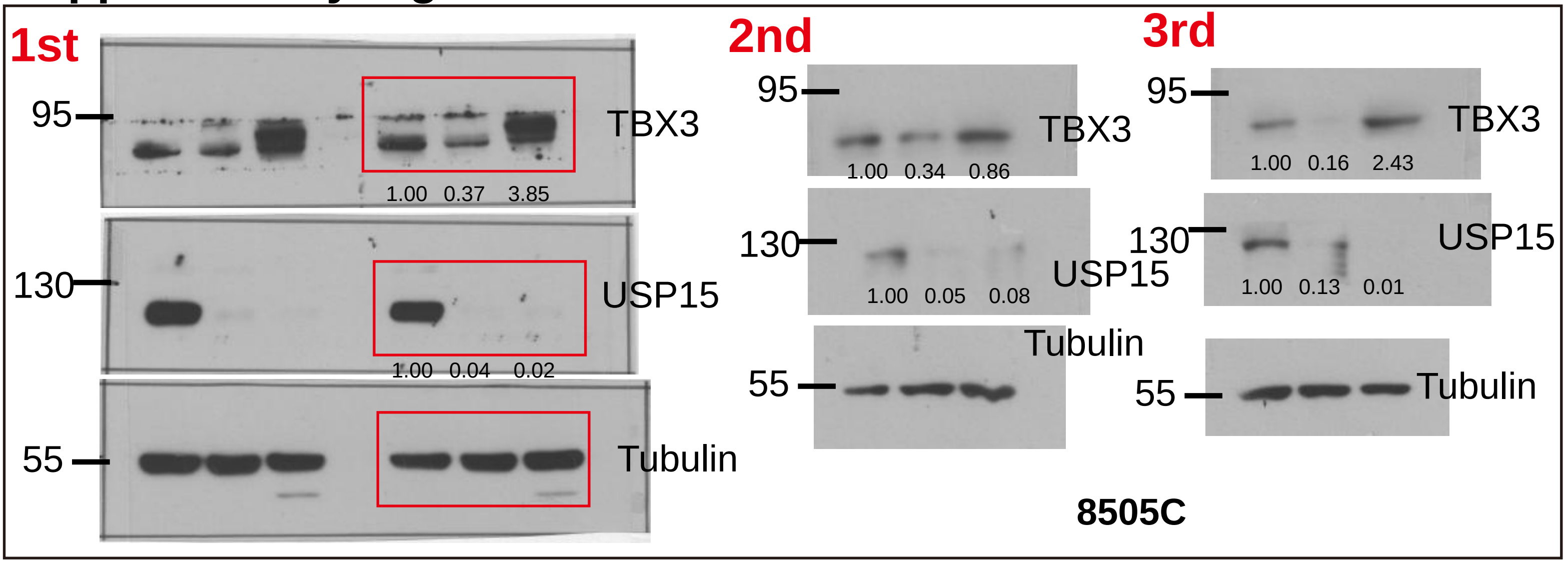

Supplementary Fig.2i

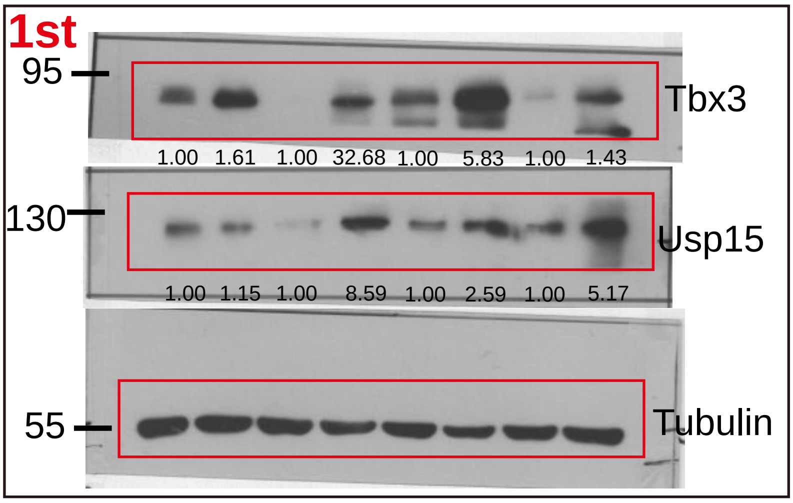

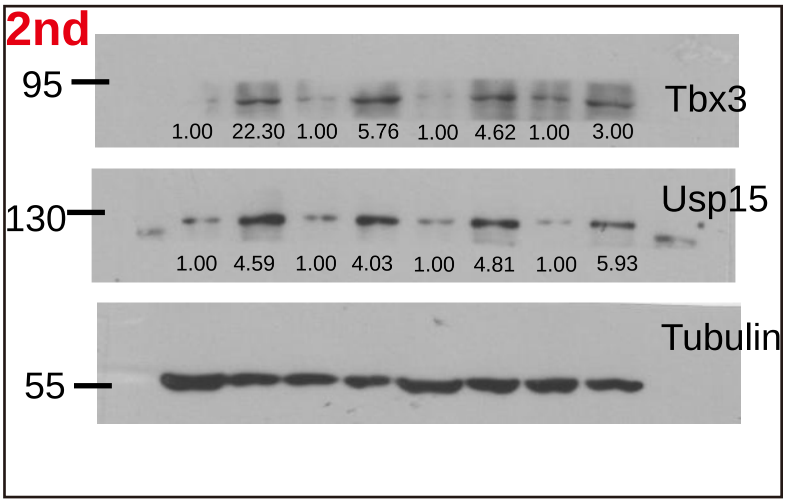

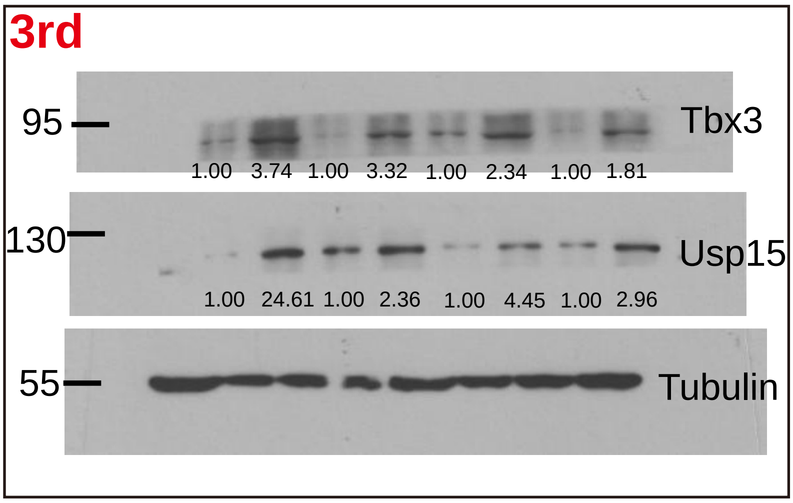

Supplementary Fig.2j

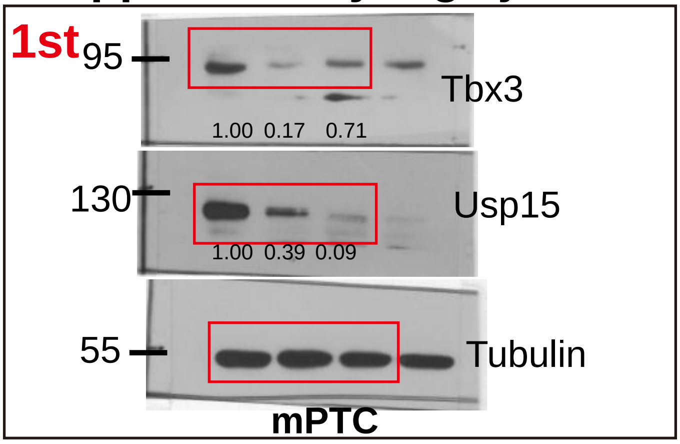

Supplementary Fig.2j

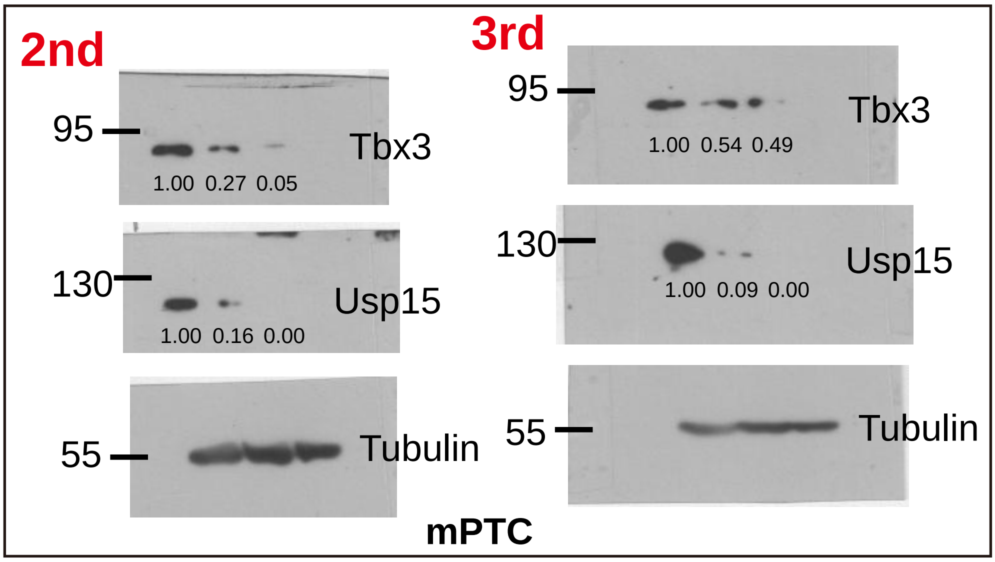

Supplementary Fig.4b

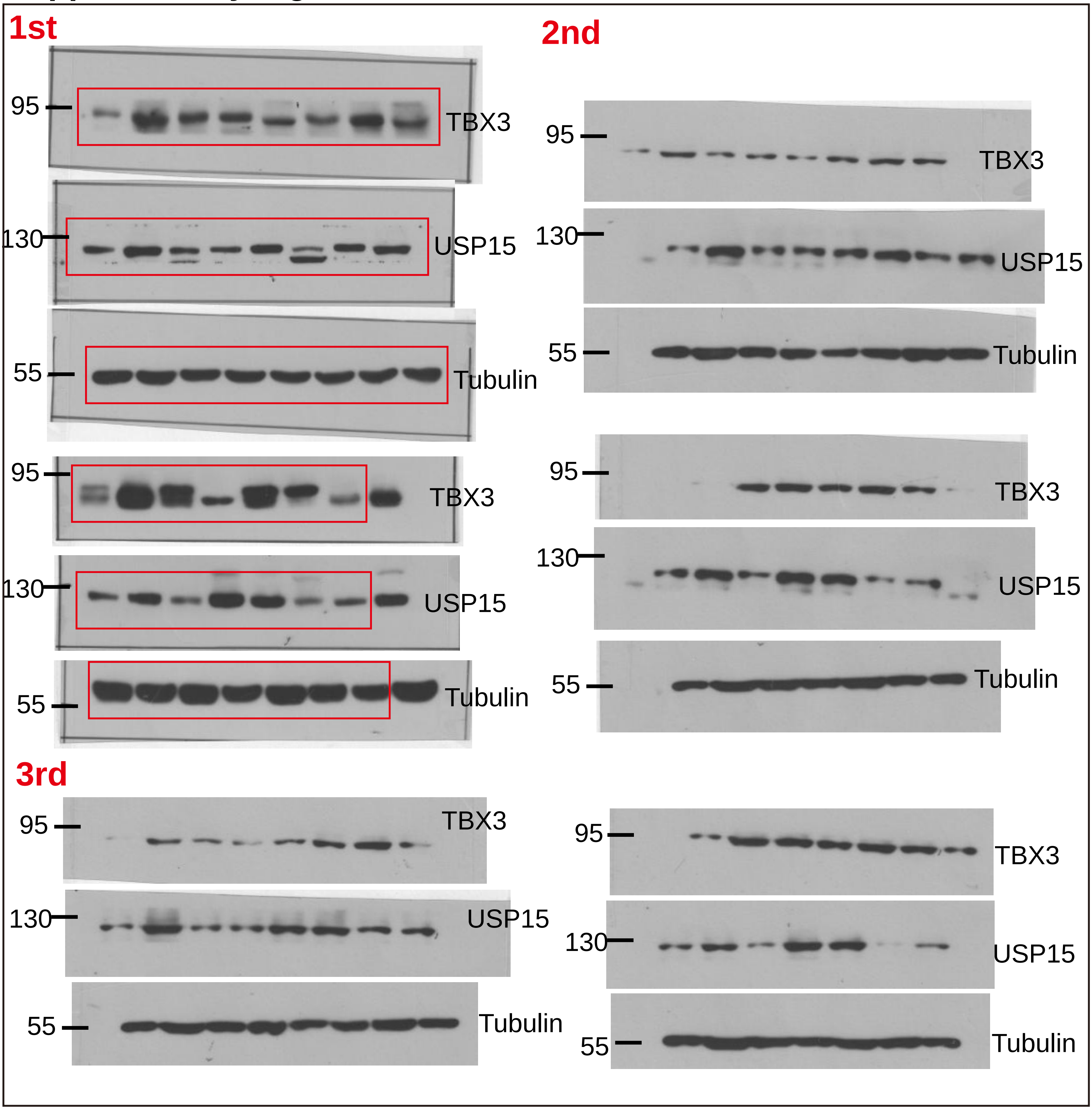

Supplementary Fig.4c

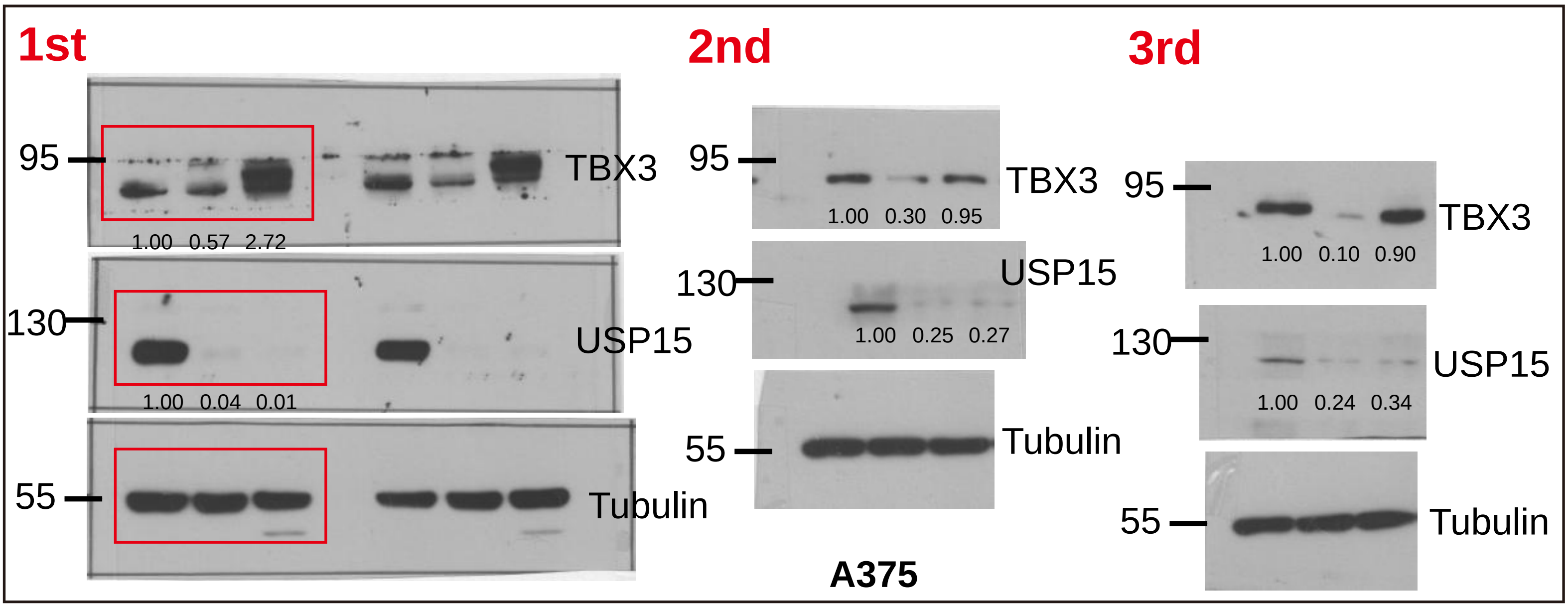

Supplementary Fig.4f

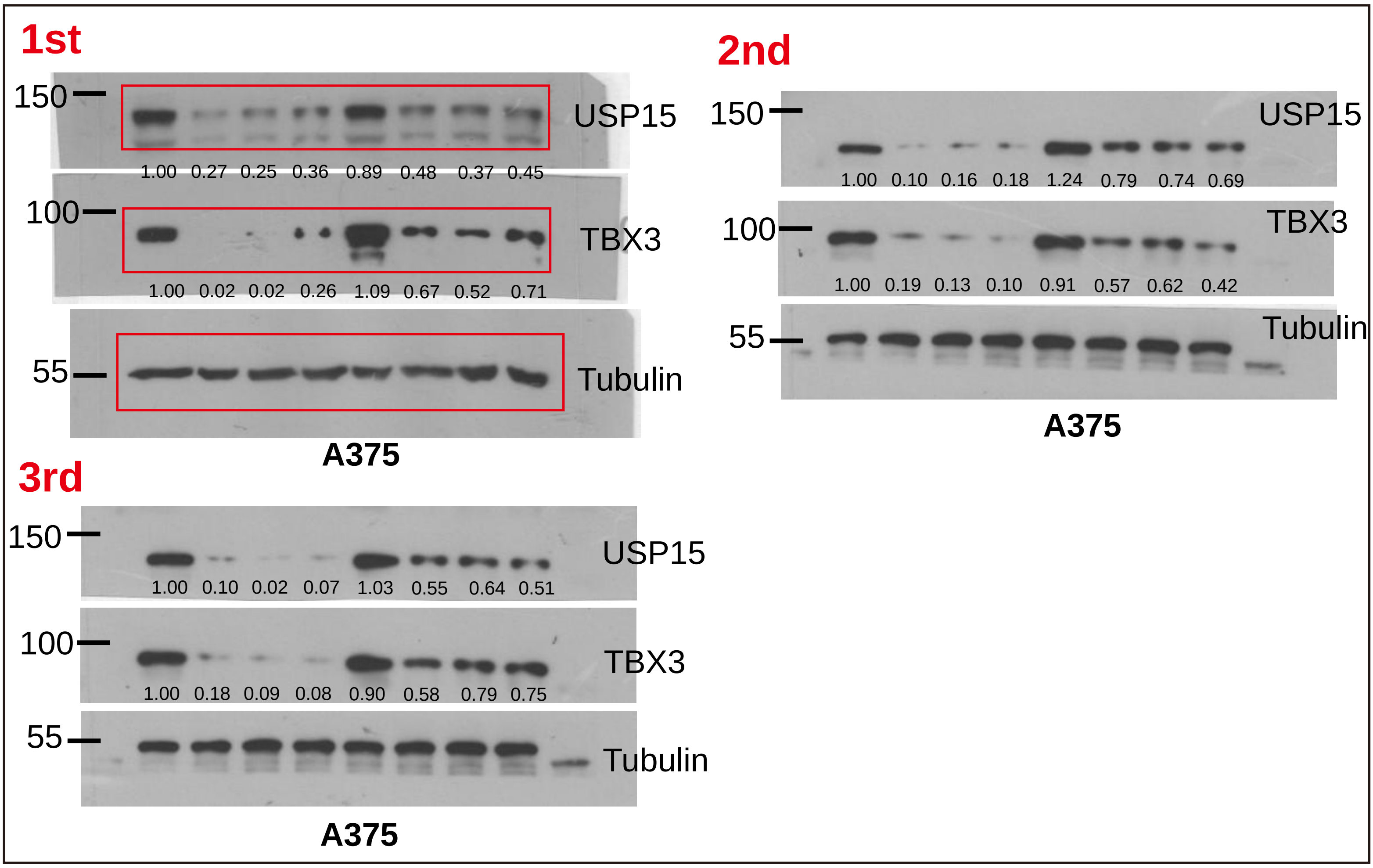

Supplement: Supplementary file 11 — Source Data [file 41467_2024_48173_MOESM11_ESM.zip › Source Data/Uncropped Blots.pdf]
